# Population Structure and Genetic Diversity in a Rice Core Collection (*Oryza sativa* L.) Investigated with SSR Markers

**DOI:** 10.1371/journal.pone.0027565

**Published:** 2011-12-02

**Authors:** Peng Zhang, Jinquan Li, Xiaoling Li, Xiangdong Liu, Xingjuan Zhao, Yonggen Lu

**Affiliations:** State Key Laboratory for Conservation and Utilization of Subtropical Agro-Bioresources, South China Agricultural University, Guangzhou, China; University of Toronto, Canada

## Abstract

The assessment of genetic diversity and population structure of a core collection would benefit to make use of these germplasm as well as applying them in association mapping. The objective of this study were to (1) examine the population structure of a rice core collection; (2) investigate the genetic diversity within and among subgroups of the rice core collection; (3) identify the extent of linkage disequilibrium (LD) of the rice core collection. A rice core collection consisting of 150 varieties which was established from 2260 varieties of Ting's collection of rice germplasm were genotyped with 274 SSR markers and used in this study. Two distinct subgroups (i.e. SG 1 and SG 2) were detected within the entire population by different statistical methods, which is in accordance with the differentiation of *indica* and *japonica* rice. MCLUST analysis might be an alternative method to STRUCTURE for population structure analysis. A percentage of 26% of the total markers could detect the population structure as the whole SSR marker set did with similar precision. Gene diversity and MRD between the two subspecies varied considerably across the genome, which might be used to identify candidate genes for the traits under domestication and artificial selection of *indica* and *japonica* rice. The percentage of SSR loci pairs in significant (*P*<0.05) LD is 46.8% in the entire population and the ratio of linked to unlinked loci pairs in LD is 1.06. Across the entire population as well as the subgroups and sub-subgroups, LD decays with genetic distance, indicating that linkage is one main cause of LD. The results of this study would provide valuable information for association mapping using the rice core collection in future.

## Introduction

Rice (*Oryza sativa* L.) feeds more than 50% of the world's population and is one of the most important crops in the world. Rice genetic resource is the primary material for rice breeding and makes a concrete contribution to global wealth creation and food security. China is well known as an origin center of cultivated rice, with abundant rice genetic resources. As early as 1920–1964, Ying Ting, An academician of Chinese academy of science, had collected more than 7128 rice landrace from all over China as well as some main rice cultivated countries. The collection is one of the earliest collections for rice germplasm resources in China and was named as Ting's collection [1]. Rice landrace contain greater genetic diversity than elite cultivars and represent an intermediate stage in domestication between wild and elite cultivars [2]. Mining the elite genes within these rice landrace is of importance to the genetic improvement of cultivated rice.

Association mapping has been proved to be an effective approach to connect structural genomics and phenomics in plants [3], [4], [5], therewith provides a promising method to mine the elite genes in germplasm resources. Association mapping has been widely used in plant research, e.g. maize, rice, barley, durum wheat, spring wheat, sorghum, sugarcane, sugar beet, soybean, grape, forest tree species and forage grasses [6].

Population used in association mapping should posses as many phenotypes as possible [7]. One of the methods to obtain most of the phenotypes is to construct the core collection. A core collection is a subset chosen to represent the most genetic diversity of an initial collection with a minimum of redundancies [8], [9], [10]. Construction of core collection was widely applied in rice as well as in other crops [11]. In our lab, a rice core collection consisting of 150 accessions based on 48 phenotypic data from 2262 accessions of Ting's collection has been constructed [1]. The abundant variation of the rice core collection provides an important reservoir of genetic diversity and potential sources of beneficial alleles for rice breeding. However, to our knowledge, no earlier research is available to apply association mapping in a core collection, which might be due to lack of information on the population structure and LD of the core collection populations.

Population structure is an important component in association mapping analysis because it can reduce both type I and II errors between molecular markers and traits of interest in an inbreeding species [12], [13], [14], [15], [16], [17]. The presence of subpopulations can result in spurious associations due to confounding of unlinked markers with phenotypic variation [18]. Low level of LD could lead to impractical whole-genome scanning because of the excessive number of markers required [19]. Also, the resolution of association studies in a test sample depends on the structure of LD across the genome [3]. Therefore, information about the population structure and extent of LD within the population is of fundamental importance for association mapping [20].

Several previous researches on rice population structure have been reported. Five major groups, i.e. *indica*, aus, aromatic, temperate *japonica*, and tropical *japonica* were detected in a sample of 234 rice varieties [21]. Eight subpopulations were found corresponding to major geographic regions among 103 rice accessions [16]. Seven subpopulations were detected within rice landrace in Guizhou province, China [22]. Two subgroups including *indica* and *japonica* as well as six sub-subgroups were found within a primary rice core collection [23]. Seven subgroups were found within a 416 rice population [24]. The varied numbers of subgroup might be due to different methods, different numbers of marker, different rice populations applied in population structure examination, which should be further studied. However, as far as we know, no information on the population structure of a rice core collection assessed with a large SSR marker set was available. Furthermore, no information is available on the number of SSRs required for such analyses.

Various methods have been proposed for examining population structure. One of the most frequently used approaches is a model-based approach STRUCTURE [25]. Principal component analysis (PCA) and principal coordinate analysis (PCoA) are also frequently used for uncovering population structure [26], [27]. Laplacian eigenfunctions (LAP) were recently reported to describe population structure [28]. Another model-based approach, MCLUST, was reported to predict population structure without genetic assumptions [29]. Despite that advantages and disadvantages of the different methods are known, few empirical comparisons are available based on a large SSR marker set. Furthermore, the Cheng's index method could discriminate *indica* and *japonica* rice based on six morphological traits, i.e. glume hair, phenol reaction, length of 1^st^–2^nd^ rachis internode, glume color at heading, leaf hair, and grain length/width [30], [31]. However, as far as we know, neither previous research on using morphological traits in population structure examination nor the comparison between morphological markers and the molecular markers in such research was available.

Two distinct subspecies, i.e. *indica* and *japonica*, existed in cultivated rice, which adapted to different ecological environments [32]. Due to the partial sterility of *indica*-*japonica* F_1_ hybrids [33], gene flow between them is difficult. The relatively independent development of these two subspecies through domestication and breeding might have resulted in divergent genomic variation. Such signatures of selection [34] might help to identify the genes underlying phenotypic variation between them as well as exploring the essence of *indica*-*japonica* hybrid vigor. However, such information is not available for rice.

For the LD within rice populations, high population structure and significant LD surrounding the *Xa5* locus was observed between sites up to 100 kb apart [35]. LD was observed to decay at 1 cM or less in rice investigated with DNA sequences [36], [37], [38]. LD decayed at 20–30 cM using SSR markers [16], [17]. Intra-chromosomal LD decayed at an average of 25–50 cM in different subgroups [24]. These studies suggest that the extent of LD varies among different genomic regions and among different rice populations examined. However, to our knowledge, no earlier research is available on the LD of a rice core collection with extensive genome-wide distributed SSR markers.

SSR markers are widely used in rice genetic for its advantages of abundance in rice genome, co-dominance, a high polymorphism rate [39]. Furthermore, using 359 SSRs and 8244 SNPs for detecting the population structure of 1537 maize accessions, Van Inghelandt et al. showed that the population structure was consistent based on SSRs and SNPs. Furthermore, SSR marker has their own advantages as compared to SNP marker respected to population genetics [40].

The objective of this study were to (1) examine the population structure of a rice core collection; (2) investigate the genome-wide distribution of genetic diversity within a rice core collection; (3) identify the extent of LD within a rice core collection with 274 genome-wide distributed SSR markers.

## Materials and Methods

### Plant material

A core collection consisting of 150 rice varieties (Table S1) was used in this study, which were mainly collected from 20 different provinces of China as well as from North Korea, Japan, Philippines, Brazil, Celebes, Java, Oceania, and Vietnam.

### Classification of *indica* and *japonica*

The *indica* and *japonica* characteristic for each rice variety was identified by the Cheng's index method. Using this method, six morphological traits, i.e. glume hair, phenol reaction, length of 1^st^–2^nd^ rachis internode (cM), glume color at heading, leaf hair, and grain length/width, were examined and scored at five levels according to Table 1. The Cheng's index was calculated by summing up of the scores of all the six traits. When the Cheng's index for a variety is 1–7, 8–13, 14–17, or 18–24, it was classified as typical *indica, indica*-clined, *japonica*-clined, or typical *japonica* rice, respectively.

**Table 1:** The morphological traits and their scoring standards of the Cheng's index method.

Traits	0	1	2	3	4
Glume hair	Short, uniform,hard and even	Hard, slightlyuniform, a little long	Medium long,less uniform	Long, a little softand not uniform	Long,crazyand soft
Phenol reaction	Black	Brown black	Grey	A little stainedalong side	Not stained
Length of 1^st^–2^nd^rachis internode (cm)	<2.0	2.1–2.5	2.6–3.0	3.1–3.5	>3.5
Glume color at heading	Grenn white	White green	Yellow green	Light green	Green
Leaf hair	Very much	Much	Moderate	Little	None
Grain length/width	>3.5	3.5–3.1	3.0–2.6	2.5–2.1	<2.0

Cheng's index is the sum of the scores of all the six traits. The Cheng's index for typical *indica* rice is between 1–7, *indica*-clined rice between 8–13, *japonica*-clined rice 14–17, and typical *japonica* rice between 18–24.

### SSR markers

274 simple sequence repeats (SSRs) distributed on the 12 chromosomes of rice were applied in this study. A total of 23, 25, 24, 22, 21, 22, 21, 25, 23, 24, 23, and 21 of these markers map to chromosomes 1 to 12, respectively. The average distance between the loci in chromosomes 1 to 12 is 7.5 cM, 8.2 cM, 9.4 cM, 7.4 cM, 7.1 cM, 6.3 cM, 5.8 cM, 5.4 cM, 5.2 cM, 4.7 cM, 5.6 cM and 5.3 cM, respectively.

### SSR array

DNA was extracted using modified SDS method [41]. The volume of the PCR reaction system was 10 µl. The profile of PCR program is: 94°C for 5 mins; 29 cycles of 94°C for 1 min, 55°C for 1 min, 72°C for 1 min; and a 5 mins final extension at 72°C. Amplified products were size separated by 6% polyacrylamide gel electrophoresis and detected by silver staining [42]. Alleles were mainly detected by BIO Imagine System and software Genetools from SynGene and by manually re-checked twice. A standard marker (100–600 bp, produced by Shanghai Biocolor BioScience & Technolgy Company) was added on each gel as control when running gel. The length of each allele was compared to the standard bands of the standard marker and scored.

### Data analysis

#### Genetic diversity

Genetic diversity was assessed using the program POWERMARKER V3.25, measured by number of alleles per locus, gene diversity, and polymorphism information content (PIC). Gene diversity and coefficient of gene differentiation among populations (Gst) [43] and the modified Rogers distance (MRD) [44] were calculated using the software gdiversity (Xu HM unpublished). Furthermore, a genome-wide distribution of gene diversity was calculated for the *indica* and *japonica* rice for each marker separately. Similarly, MRD between *indica* and *japonica* rice was calculated on an individual marker basis.

#### Structure analysis

Software STRUCTURE V2.3.1 was applied to infer historical lineages that show clusters of similar genotypes [25], [45], [46], [47]. Due to the distribution of L(*K*) did not show a clear cutoff point for the true *K*, an ad hoc measure Δ*K* [45] was used to detect the numbers of subgroup. The membership of each genotype was run for the range of genetic clusters from value of *K* = 1 to 15 with the admixture model, and for each K it was replicated 5 times. Each run implemented with a burn-in period of 100,000 steps followed by 100,000 Monte Carlo Markov Chain replicates [47]. That run with the maximum likelihood was applied to subdivide the varieties into different subgroups [48] with the membership probabilities threshold of 0.80 as well as the maximum membership probability among subgroups. Those varieties with less than 0.80 membership probabilities were retained in the admixed group (AD). The results from STRUCTURE were displayed by DISTRUCT software [46].

The allele frequencies at each marker and for each variety were calculated and used for PCA analyses [26]. PCoA [27] based on MRD estimates between pairs of varieties was performed. In addition, LAP [28] was used to reveal the population structure, where the threshold of correlation coefficients *eps* was set to 0.8. Finally, the model-based approach MCLUST was used to determine the number of subgroups as well as to provide the membership probabilities [29]. MCLUST was performed on all the SSR marker genotypes as well as the Cheng's index and the six morphological traits of Cheng's index. Models for 1 to 30 subgroups were examined. The correspondence between the inbreds' assignment by MCLUST and STRUCTURE and the germplasm type information were compared.

In order to determine the number of SSRs required to detect the underlying population structure, a resampling analysis was performed. In each of 100 repetitions, subsets of the markers (12 to 274 by 12 grad) were either randomly selected (random sampling) or sampled in such a way that the selected markers were equally distributed across the genome (stratified sampling) [40]. MCLUST analysis was performed on the selected marker genotypes. The correspondence between the varieties' assignment by MCLUST based on the entire set of 274 SSRs and different resampling subsets was compared. The MRD was calculated for each pair of varieties based on the selected SSR markers and the coefficient of variation (CV) across all 100 repetitions was calculated.

Software NTSYS was applied to construct the neighbor-joining tree on the basis of similarity measures [49] (Numerical Taxonomy and Multivariate Analysis System, 1997). Software MEGA V4.0 was used to observe the NJ tree [50]. A clustering tree was generated using PROC CLUSTER in SAS 9.0 based on the Cheng's index and the scores of its six traits (SAS Institute 2002).

#### LD analysis

The level of LD between pairs of locus was performed using the software TASSEL V2.1 (http://www.maizegenetics.net/). If within a chromosome region all pairs of adjacent loci were in LD, this region was referred to as a LD block [20].

If not stated differently, all analyses were performed with the statistical software R (R, Development Core Team, Vienna, Austria, 2011).

## Results

With the Cheng's index criterion, 32 varieties were classified to *japonica* rice, among which 24 varieties were typical *japonica* rice and 8 varieties *japonica*-clined rice (Table S1). Similarly, 118 varieties were classified to *indica* rice, among which 16 varieties were *indica*-clined rice and 102 varieties typical *indica* rice. The clustering analysis based on the Cheng's index and the scores of the six morphological traits revealed two clusters when the Ward's distance was 4 (Figure S1). The *indica* and *japonica* varieties located in two different clusters.

**Figure 1:**
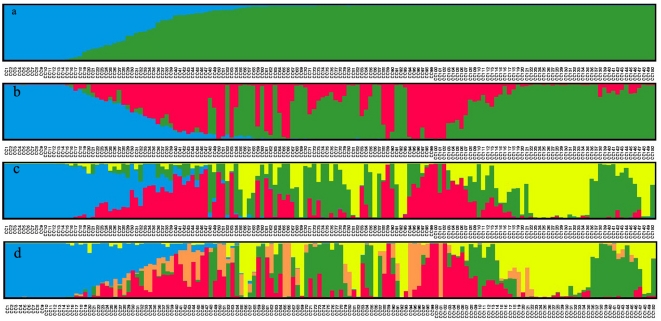
Membership probability of assigning genotypes of the entire population to (**a**) **two,** (**b**) **three,** (**c**) **four,** (**d**) **five subgroups.** The height of each bar represents the probability of varieties belonging to different subgroups. The varieties were sorted according to their membership probability in (a).

**Figure 2:**
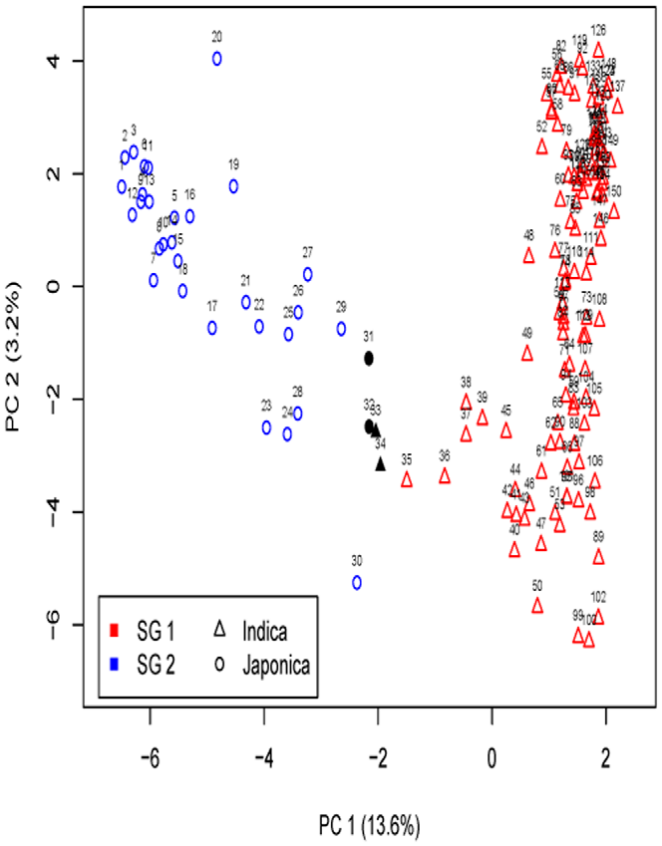
Principal component analysis on SSR genotypes of the entire population. PC 1 and PC 2 refer to the first and second principal components, respectively. The numbers in parentheses refer to the proportion of variance explained by the corresponding axes. Symbols identify the germplasm types and colors the STRUCTURE and MCLUST subgroups. SG 1 and SG 2 are the two subgroups identified by both MCLUST on SSR marker genotypes and STRUCTURE assigned with the max membership probability. The black dots are the varieties with different assignment by MCLUST and STRUCTURE. I and J are *indica* and *japonica* rice identified by Cheng's index.

The log likelihood revealed by STRUCTURE increased gradually from *K* = 1 to *K* = 15 and shoed no obvious optimum (data not shown). In contrast, the maximum of the ad hoc measure Δ*K* was observed for *K* = 2 (Figure S2a), which indicated that the entire population could be divided into two subgroups (i.e. SG 1 and SG 2). With the membership probabilities of ≧0.80, 111 *indica* varieties were assigned to SG 1, 20 *japonica* varieties to SG 2 and 19 varieties were retained to the AD (Figure 1, Figure S3a). Compared to the *indica*-*japonica* classification by Cheng's index method, SG 1 is consisted of 102 typical *indica* rice and 9 *indica*-clined rice (Table 2, Figure S3). SG 2 is consisted of 20 typical *japonica* rice. AD is consisted of 7 *indica*-clined rice, 8 *japonica*-clined rice and 4 typical *japonica* rice. Furthermore, with the criterion of maximum membership probabilities among the subgroups, 118 *indica* rice and one *japonica* rice were assigned to SG 1 and 31 *japonica* rice were assigned to SG 2 (Figures 1 and 2). The assignment by STRUCTURE showed for 99.33% of the varieties correspondence with the germplasm type information (i.e. the *indica* and *japonica* types) (Table 3). When the number of subgroups increased from two to five, the varieties in SG 1 could be further assigned to different sub-subgroups, but it was not true for the SG 2 (Figure 1).

**Table 2:** Distribution of the rice varieties in the entire collection, subgroups, and sub-subgroups identified by STRUCTURE.

Group	*N*	Definition	Predefined taxonomic identities							
			*J*	*I*	LS	ES	Te	Tr	ST	Missing
**Entire core collection**	150		32(21%)	118(79%)	65(44%)	83(55%)	28(19%)	4(3%)	116(77%)	2
**SG 1**	111		0	111(100%)	57(51%)	53(48%)	9(8%)	3(2%)	99(89%)	1
**SG 1a**	24	*I*-IS	0	24(100%)	12(50%)	12(50%)	6(25%)	0	18(75%)	0
**SG 1b**	34	*I*-ST	0	34(100%)	15(44%)	19(56%)	8(23%)	3(9%)	23(68%)	0
**SG 1c**	21	*I*-LS	0	21(100%)	21(100%)	0	0	0	21(100%)	0
**SG 1d**	32	*I-ES*	0	32(100%)	7 (22%)	25(78%)	1(3%)	0	31(97%)	0
**SG 2**	21		21(100%)	0	4(19%)	16(76%)	12(57%)	0	8(38%)	1
**AD**	18		11(61%)	7(39%)	6(33%)	12(67%)	6(33%)	3(17%)	9(50%)	0

Note: *I*-*Indica*, *J*-*Japonica*, ES-Early seasonal, LS-Late seasonal, Te-Temperate, Tr-Tropical, ST-Subtropical. Proportion is indicated in parenthesis, where *N* is the sample size.

**Table 3:** Correspondence of the assignments by different methods and the germplasm types.

	MCLUST_M	MCLUST_C	Germplasm type
Structure	97.33%	96.00%	99.33%
MCLUST_M		97.33%	98.00%
MCLUST_C			96.67%

Structure is the assignment by STRUCTURE software, MCLUST_M is the assignment by MCLUST based on the SSR marker genotypes, while MCLUST_C is the assignment by MCLUST based on the six morphological traits of Cheng's index as well as Cheng's index. Germplasm type (i.e. *indica* and *japonica* rice) is identified by Cheng's index (See Materials and Methods).

Due to that SG 1 is consisted of a large amount of rice varieties, an independent STRUCTURE run was performed for the subgroup. Δ*K* showed its maximum value for *K* = 4 (Figure S2b), which indicated that four sub-subgroups existed in the SG 1 (i.e. SG 1a-SG 1d) which consisted of 24, 34, 21, and 32 varieties, respectively (Table 2). The differentiation in early-seasonal or late-seasonal rice and different original regions contributed to the sub-subgroup's population structure.

**Figure 3:**
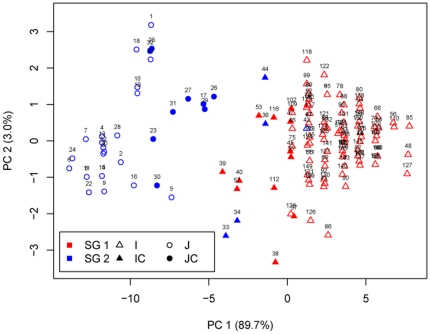
Principal component analysis on the six phenotypic traits of Cheng '**s index as well as Cheng's index of the entire population.** PC 1 and PC 2 refer to the first and second principal components, respectively. The numbers in parentheses refer to the proportion of variance explained by the corresponding axes. Symbols identify the germplasm types and colors the MCLUST subgroups. SG 1 and SG 2 are the two subgroups identified by MCLUST on the six phenotypic traits of Cheng's index as well as Cheng's index. I, IC, J, JC are *indica*, *indica*-clined, *japonica*, *japonica*-clined rice.

PCA, PCoA, as well as LAP based on the marker genotypes revealed two distinct clusters for the entire population (Figure S3), which is related to their germplasm types. The first and second principal component explained 13.6% and 3.2% of the molecular variance, respectively. The first two principal coordinates explained 13.7% and 3.3% of the molecular variance. In addition, the first and second lapvectors of LAP explained 11.0% and 2.4% of the molecular variance, respectively. Furthermore, PCA based on the Cheng's index and the scores of the six morphological traits also revealed two clusters (Figure 3). The first and second principal component explained 89.7% and 3.0% of the phenotypic variance, respectively.

**Figure 4:**
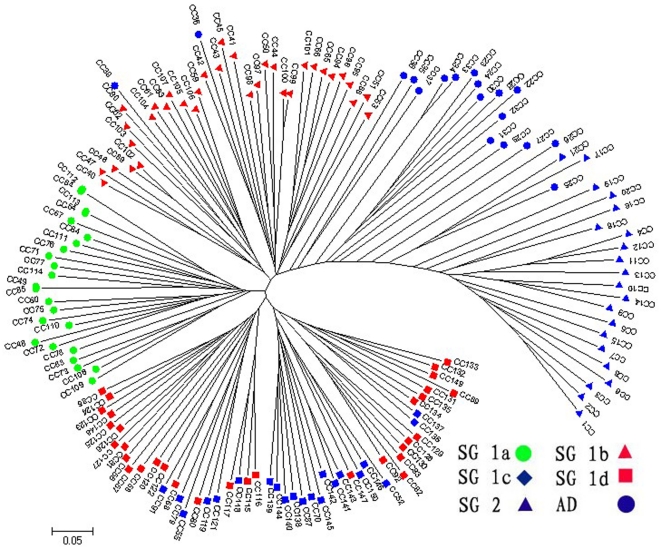
Unrooted neighbor-joining trees of 150 rice varieties in the core collection.

The number of subgroups 1–30 was examined by different models of MCLUST based on the SSR marker genotypes as well as the Cheng's index and the scores of the six morphological traits. The Bayesian information criterion revealed two subgroups for both cases (Figure S4). MCLUST analysis based on the SSR marker genotypes as well as based on the Cheng's index and the scores of the six morphological traits showed for 98.00% and 96.67% of the varieties correspondence with the germplasm type information (Table 3, Figures 2 and 3). They also showed more than 96.00% of assignment correspondence with each other as well as with the assignment by STRUCTURE. Furthermore, neighbor-joining tree (NJ) showed six branches within the entire population, which were fairly consistent with the STRUCTURE-based membership assignment for most of the varieties (Figure 4).

**Figure 5:**
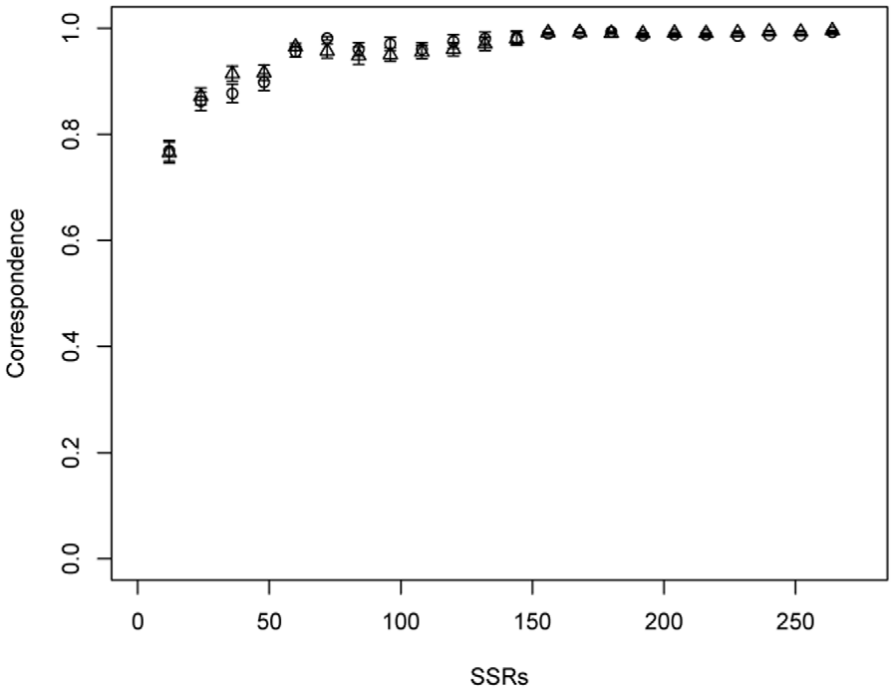
Correspondence between the assignment of all 150 rice varieties by MCLUST based on the entire set of 274 SSRs and on different subsets of SSR markers (from 12 to 274 with grads of 12) selected (**a**) **at random (triangles) or stratified (circles) with 100 replications.** The vertical lines at each point indicate the standard error. For details see Materials and Methods.

**Figure 6:**
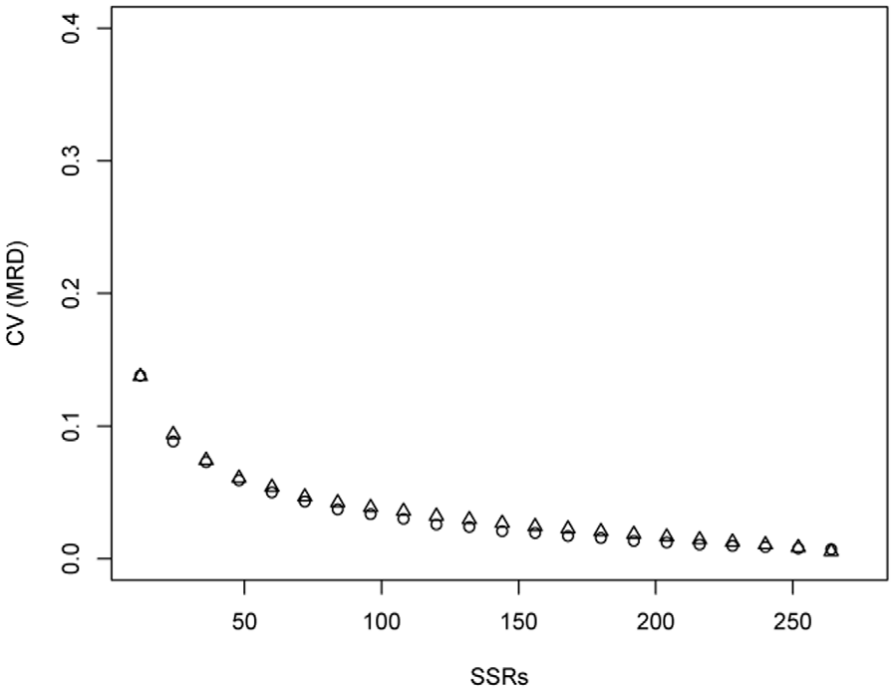
Coefficient of variation (CV) of modified Roger '**s distance (MRD) among all pairs of varieties assessed by random (triangles) and stratified (circles) resampling with 100 replications.** For details see Materials and Methods.

MCLUST was used to assign rice varieties based on different resampling subsets of all SSR markers to clusters, where the correspondence to the clustering using all SSRs improved with increasing number of SSR markers (Figure 5). When the number of SSR markers reached about 72, not much higher correspondence could be obtained by further increasing the number of SSRs. Similarly, the CV of MRD among all pairs of varieties decreased as the number of SSR markers increased (Figure 6). When the number of SSR markers reached about 72, not much lower CV of MRD could be obtained by further increasing the number of SSRs. The stratified resampling strategy revealed a slightly higher correspondence and lower CV compared to the random resampling strategy.

Abundant genetic diversity was observed within the 150 varieties of rice core collection (Table 4, Table S2). 1063 alleles were detected in total. The alleles ranged from 2 to 12 per locus with an average of 3.88 alleles per locus. An average of PIC was 0.4831. The average alleles per locus for *indica* and *japonica* rice were 3.71 and 3.26, respectively. There were 175 unique alleles for *indica* rice while 51 unique alleles for *japonica* rice. High MRD existed between *indica* and *japonica* rice, which was 0.443.

**Table 4:** Genetic diversity for the entire collection and different germplasm types.

Germplasm type	N	Allele per locus	Allele frequency	Unique allele	Gene diversity
Entire collection	150	3.88	0.282	-	0.544
*Indica* rice	118	3.71	0.269	175	0.484
*Japonica* rice	32	3.26	0.307	51	0.454

N is the sample size, and MRD the modified Roger's distance.

The average gene diversity of the entire population, *indica* rice, and *japonica* rice were 0.544, 0.484, and 0.454, respectively (Table 4). Gene diversity for *indica* and *japonica* rice varied across the rice genome (Figure S5). For most genome regions, the *indica* rice showed a higher gene diversity than the *japonica* rice. However, for a few regions, the opposite was true. Moreover, a different degree of divergence measured by MRD between these two germplasm types was observed across the genome (Figure S6).

**Figure 7:**
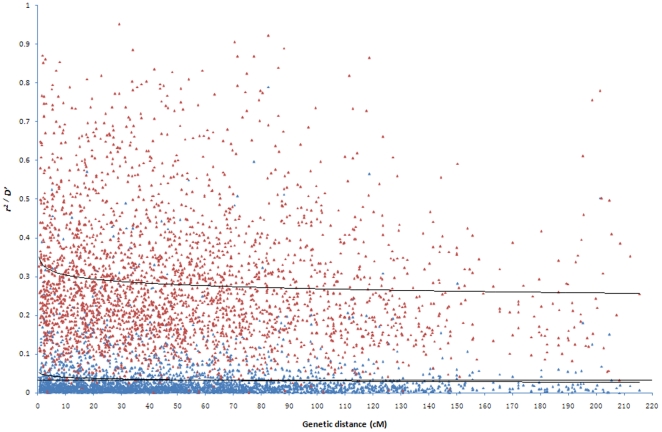
LD decay plot within the entire population. Squared correlations of allele frequencies (*r^2^*, blue triangles) and weighted standardized disequilibrium coefficient (*D*', red triangles) against genetic distance(cM) between linked loci in the entire population. The horizontal line indicates the 75th percentile of *r^2^* for unlinked loci.

Across the entire population as well as the two subgroups, both *D*' and *r^2^* decayed with genetic distance (Figure 7). Within the entire population, LD decayed to 75% quantile of *r^2^* for unlinked loci at 40–50 cM. For SG 1and SG 2, LD decayed to 75% quantile of *r^2^* for unlinked loci around the region of 3–4 cM and <2 cM, respectively (Figure S7). In addition, LD decayed to 75% quantile of *r^2^* for unlinked loci around the region of <1 cM, 2–3 cM, <1 cM and <1 cM for SG 1a-SG 1d, respectively (Figure not shown).

The percentage of SSR loci pairs in significant (*P*<0.05) LD was 46.8% in the entire population (Table 5). In the subgroups, the percentage of loci pairs in LD was lower and ranged from 5.7 to 18.8%, and the ratio linked to unlinked loci in LD varied from 1.15 to 1.27. In addition, for the sub-subgroups, the percentage of loci pairs in significant (*P*<0.05) in SG 1b was highest (11.7%), while the percentage of loci pairs in significant (*P*<0.05) in SG 1a was lowest (6.4%), where the ratio of linked to unlinked loci in LD varied between 1.21 and 1.37.

**Table 5:** Percentage of SSR loci pairs in significant (*P*<0.05) linkage disequilibrium (LD) in the rice core collection.

Group	*N*(sample size)	Loci pairs in LD (%)			Ratio linked to unlinked loci in LD
		Linked	Unlinked	Total	
**Entire core collection**	150	49.5	46.6	46.8	1.06
**SG 1**	111	21.2	18.5	18.8	1.15
**SG 1a**	24	7.9	6.2	6.4	1.27
**SG 1b**	34	15.6	11.4	11.7	1.37
**SG 1c**	21	10.0	7.5	7.7	1.33
**SG 1d**	32	11.9	9.8	9.9	1.21
**SG 2**	21	9.9	7.8	8.0	1.27
**AD**	18	6.9	5.6	5.7	1.23

The total number of LD blocks varied from 56 (the entire population) to 11 (SG 2), while the maximum average length of LD block was 7.1 cM (the entire population), and the minimum was 2.8 cM (SG 2) (Table 6).

**Table 6:** Number of linkage disequilibrium (LD) blocks per chromosome and their average length per chromosome in centiMorgan(cM) in the entire population and subgroups.

Chromosome	Group								
	Entire population		SG 1		SG 2		AD		
	Number ofblocks^a^	Length (cM)	Number of blocks^a^	Length (cM)	Number of blocks^a^	Length (cM)	Number of blocks^a^	Length (cM)	
**1**	8	2.9	7	4.5	1	3.2	1	19.6	
**2**	8	5.9	3	8.1	0	0	4	5.1	
**3**	3	9.6	6	2.1	2	2.4	0	0	
**4**	2	7.9	5	8.1	1	1.4	2	13.9	
**5**	3	11	0	0	1	6.5	1	1.9	
**6**	4	8.2	6	6.9	2	2.3	2	2.3	
**7**	5	7.9	3	11.2	1	2.2	0	0	
**8**	4	14.4	3	2.4	1	8.1	0	0	
**9**	3	4.6	1	0.8	0	0	2	5.8	
**10**	8	5.4	3	13.4	1	6.9	1	1.1	
**11**	6	3.8	1	2.9	0	0	3	6.1	
**12**	2	3.3	1	3.8	1	3.8	0	0	
**Sum**	56		39		11		16		
**Mean**		7.1		5.4		2.8		4.7	

Note: ^a^ An LD block consists of a sequence of markers for which all pairs of adjacent loci are in significant (*P*<0.05) LD.

## Discussion

### Comparisons of different approaches in population structure examination

Knowledge about the patterns of population structure is essential for efficient germplasm organization. A model-based approach implemented in the software STRUCTURE might be the most frequently used method. In this study, the method successfully detected two subgroups in the entire population and assigned the rice varieties to the two subgroups. Furthermore, the assignment by STRUCTURE has the highest correspondence (99.33%) with the germplasm type information compared to other methods (Table 3, Figure 2), where only one *japonica*-clined rice (CC32) was mis-assigned because it is close to *indica*-clined rice. However, the high computational requirements of STRUCTURE analyses for large dataset hindered its application [51]. Instead, we applied PCA, PCoA, as well as LAP to reveal the population structure. These methods could well graphically show two distinct clusters for the entire population (Figure S3), which is high related to the known germplasm type information as well as the STRUCTURE subgroups. These methods have neither computation burden nor assuming any population genetic model [26], [27]. However, they don't provide the information on the number of subgroups and assignment of individuals to subgroups.

MCLUST, implemented in a R package, could determine the numbers of subgroup as well as the cluster membership probability simultaneously without genetic assumptions [29]. MCLUST analysis based on the SSR marker genotypes revealed two subgroups in the entire population (Figure S4) and had a high correspondence (98.00%) of assignment with the known germplasm type information (Table 3). The method also had a 97.33% of assignment correspondence with the assignment by STRUCTURE, where the former method can assign the *japonica*-clined variety CC32 correctly but mis-assigned one *japonica*-clined variety (CC31) and two *indica*-clined varieties (CC33 and CC34). Another advantage of MCLUST is that it can provide distinct membership probability to undoubtedly assign admixed individuals to subgroups. For example, the membership probability of variety CC32 calculated by MCLUST were 1 and 1.07×10^−49^ in the SG 1 and SG 2, respectively, while they were 0.517 and 0.483 calculated by STRUCTURE.

Furthermore, as far as we know, this study was the first time to attempt to use morphological traits to reveal the rice population structure, which can be fulfilled by MCLUST analysis. Based on the scores of the six morphological traits and Cheng's index, MCLUST revealed the population structure of two subgroups as the MCLUST analysis on the SSR marker genotypes did (Figure S4). Moreover, the assignment by the method has a high correspondence (96.67%) with the germplasm type information and >96% high correspondence with the assignment of STRUCTURE and MCLUST based on the SSR marker genotypes (Table 3, Figure 3). The population structure was also confirmed by both the clustering analysis and PCA based on the Cheng's index and the scores of the six morphological traits (Figure 3, Figure S2). Due to that the cost for the detection of six morphological markers of Cheng's index is rather lower than that of SSR markers, MCLUST based on the morphological markers might be the cheapest one to detect population structure in our case. However, the precision will be lower compared to other methods.

High correspondence was also shown between the assignment of MCLUST and STRUCTURE based on SNP markers with known germplasm type information of sugar beet [52]. However, in that research, MCLUST was performed indirectly on the principal components, principal coordinates, or lapvectors instead of SNP genotypes and the number of subgroups was difficult to be determined. The reason might be due to that SSR markers are multi-allelic while SNP markers bi-allelic thus more information was provided by SSR than SNP markers. As the method is not computationally intensive, it might be valuable alternative for detecting population structure.

### Population structure of the rice core collection

The results of the STRUCTURE analysis revealed the presence of two subgroups in the entire core collection (Figure S2). This observation was in accordance with the clustering observed in the PCA, PCoA, LAP, and clustering analyses as well as with the MCLUST analysis and with the *indica*-*japonica* classification (Table S1, Figureŝg S1, S3 and S4). With the criterion of maximum membership probabilities among the subgroups, 118 *indica* rice and one *japonica* rice were assigned to SG 1 and 31 *japonica* rice were assigned to SG 2 (Figure 1 and 2). The mis-assigned one (CC32) is a *japonica*-clined rice and has a close relationship with *indica*-clined rice (Figure 2). Similar population structure was observed in previous research [23], where two subgroups (*indica and japonica*) were found within a primary core collection of 3,024 rice landraces in China. The results indicated that *indica*-*japonica* differentiation might be the main cause for population structure in the core collection. The reason might be due to that (i) strong reproductive barriers existed in the *indica*-*japonica* hybrids which hindered the gene flow between them; (ii) the two subspecies are adapted to different ecological enviroment, for example, in China, *indica* rice is cultivated in south and central China whereas *japonica* rice in North China or the high latitude regions, thus has less chance to be exchanged.

Furthermore, with the membership probabilities of 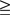 0.80, 111 *indica* varieties (102 typical *indica* rice and 9 *indica*-clined rice) were assigned to SG 1, 20 typical *japonica* varieties to SG 2, and 19 varieties were retained to the AD (Figure 1, Figure S3a). The AD was consisted of 7 *indica*-clined rice, 8 *japonica*-clined rice and 4 typical *japonica* rice. The neighbor-joining tree showed that these varieties located between *indica* and *japonica* branches (Figure 4). The results of PCA, PCoA, and LAP also showed that these varieties located between typical *indica* and *japonica* rice (Figure S3). These varieties might be the intermediate types between *indica* and *japonica* rice and have high compatibility with both *indica* and *japonica* rice, which would be valuable germplasm resources to the utilization of hybrid vigor of *indica*-*japonica* hybrid.

Further independent STRUCTURE run on SG1 indicated that SG 1 could be subdivided into four sub-subgroups (i.e. SG 1a-SG 1d), which consisted of 24, 34, 21 and 32 varieties, and corresponded to intermediate seasonal *indica*, sub-tropical *indica*, late seasonal *indica* and early seasonal *indica*, respectively (Table 2). This result was consistent with its neighbor-joining (Figure 4). Similar population structure was observed by other researches, for example, five major groups were detected within a diverse sample of 234 rice varieties among which included *indica, aus, tropical japonica, temperate japonica and aromatic* [21], and six sub-subgroups (*japonica* lowland, *japonica* upland, *japonica* medium, *indica* early, *indica* late and *indica* medium) were observed within a primary core collection of 3,024 rice landraces in China, which indicated that *indica* was more clearly subdivided by seasonal ecotypes [23].

### Comparison of marker numbers for population structure analysis

The correspondence of assignment by MCLUST based on different subsets of 12–274 SSRs by 12 grad vs. the whole 274 SSR markers were compared (Figure 5). The correspondence improved with increasing SSR markers. When the number of SSRs reached about 72, the correspondence of the selected SSR subset vs. the whole SSR set was more than 95% and reached a plateau. Similarly, the CV of MRD estimates among all pairs of varieties decreased with increasing SSR markers (Figure 6). This is due to the fact that a high number of molecular markers provides a high precision for determining population structure as well as for measuring the genetic distance between inbreds [52]. When the number of SSR markers was about 72, the change trend reached a plateau and not much further improvement could be obtained by further increasing the marker numbers. The stratified resampling strategy showed a little higher correspondence and a little lower CV of MRD than that of random resampling strategy. These results indicated that in the examined rice core collection about 72 SSR markers (26% of the total markers) would be required to determine the same population structure as the whole 274 SSR markers did and that this estimation would be done with a similar precision.

The percentage of SSRs predicted in our study to be required for population structure examination is equivalent to (i) the research of [52], where 100 out of 328 SNPs (30%) were required to examine the sugar beet population structure; (ii) the research of [40], where 25% of the SSRs (90 out of 359 SSRs) were required for MRD estimates with similar precision as the whole marker set did. As the costs for genotyping will also increase with an increasing number of SSR markers, our result might be a good reference to select optimal number of markers for population structure analyses as well as association mapping.

### Genetic diversity of the rice core collection

In this study, across the entire population, we observed an average number of alleles per locus of 3.88 which ranged from 2 to 12, a gene diversity of 0.544 and a PIC of 0.4831 (Table 4 and S2). The average number of alleles per locus was consistent with the result of a diverse rice population in China, while both gene diversity and PIC were higher (0.4736 and 0.4214) than the research [24]. This might be explained by that all rice varieties in the core collection of our study were selected from 2262 accessions of landraces which were collected in early times of 20th century so that more diversity might exist in such population. However, the average number of alleles per locus, gene diversity and PIC of our study are less than other reports [16], [21], in which the rice varieties were the worldwide collections.

Average gene diversity for *indica* rice and *japonica* rice was 0.484 and 0.454, respectively (Table 4). High differentiation assessed with MRD was observed between the two subspecies, which was 0.443. *Indica* rice has 175 unique alleles while *japonica* rice 51 unique alleles. The results indicated that the two subspecies are distinct from each other, which might be due to that they are developed independently. Our results showed no large difference for the average gene diversity between *indica* and *japonica* rice. The reason might be due to that the *japonica* varieties in this study were collected from wide regions including Java, Japan, Celebes, south China, north China, northeast China, Yangtze River region in China, and Taiwan while most of the *indica* rice were collected only in China (Table S1).

*Indica* and *japonica* rice have been developed independently for many years. Consequently, the genomic regions might be varied due to different selection pressure on the target genes and traits between the two subspecies. Our results indicated that gene diversity for *indica* and *japonica* rice varied across the rice genome (Figure S5). Moreover, a different degree of divergence measured by MRD between the two subspecies was observed across the genome (Figure S6).

The genome-wide distribution maps of genetic diversity might be a first step to identify the target genes or regions selected during breeding history. For example, one SSR marker RM16 has different gene diversity (0.651 for *indica* while 0.119 for *japonica*) and high MRD estimate (0.61) between the two subspecies (Figure S5, Figure S6), which was identified to be linked to the QTLs of grain length, grain width, grain length/width, and awn length by previous researches (http://www.gramene.org/). Similarly, PSM419 and RM247 located in chromosome 12 were linked to the QTL of grain width; RM71 located in chromosome 2 was linked to the QTL of grain length/width; three markers, i.e. RM252 in chromosome 4, RM6 and RM240 in chromosome 2, were linked to the QTL of leaf length. A similar previous research was shown that screening of such signature of selection could identify a panel of known genes as well as some interesting candidate genes and QTLs in Holstein cattle [34]. More genes related to the difference between *indica* and *japonica* rice might be present in the most divergent genomic regions between the two subspecies. Common genes under selection in the breeding program of the both subspecies (e.g. disease resistant genes) might be present in the genomic regions showing the same level of gene diversity and low MRD, which should be studied in future.

### Extent of LD and consequences for association mapping

For the entire population, we observed that 46.8% of the loci pairs were in significant LD (*P*<0.05), and a ratio of linked to unlinked loci in significant LD of 1.06, which was of the same order of magnitude for maize [20], [53]. The percentage of the loci pairs in significant LD was lower than that (63%) of a reported diverse rice population in China [24], which might be due to the fact that our study was based on a core collection population which might be more diverse than the former research thus the LD level was reduced. This high extent of LD between unlinked loci might be due to genetic drift, familial relatedness and population structure. Within the subgroups and sub-subgroups, the extent of LD (5.7–18.8%) was lower compared to the entire population. This finding can be explained by the fact that the less numbers of varieties in the subgroups as well as sub-subgroups than in the entire population, would lead to a reduction in power to detect LD [24], [1]. Another factor contributing to our observation was the reduced influence of population structure on LD within the subgroups.

We found that the LD decay distance were in the region of 40–50 cM in the entire germplasm set, <5 cM in the subgroups and 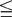
3 cM in the sub-subgroups. The LD decay distance for the entire germplasm set was in the upper limit of a reported diverse rice population [24], where LD in a set of germplasm consisting of 416 rice accessions did not decay until 25–50 cM, while it was longer than that of a world collection consisting of 92 rice accessions investigated with 123 SSR markers [16]. The difference might be due to that the rice varieties in the latter research [16] are more diverse than those in our study and fewer accessions were used. The difference might be explained that the fact that a stronger population structure might exist in a core collection than a natural population thus affected the LD level. This explanation could be supported by the fact that LD decay distance in subgroups and sub-subgroups was much shorter than that in the entire germplasm set in our study while the former showed less population structure.

The length of chromosome regions in LD is crucial for application of association mapping because (i) regions in LD need to be present in order to detect marker-phenotype associations and (ii) the length of the regions limits the resolution of association mapping [20]. The LD blocks observed in this study had an average length of 7.1 cM, and the number of LD blocks for the entire population was 56 in this study, while LD blocks were observed to have an average length of 33 cM and 22 LD blocks using 100 SSRs in maize [20]. The difference might be explained by that more SSR markers were used in our study than those in the latter research which could reduce the observed average LD block length and increase numbers of LD block.

### Conclusions

We identified based on different statistical methods two distinct subgroups within the rice core collection, which is in accordance with the differentiation of *indica* and *japonica* rice. MCLUST based on SSR marker genotypes as well as the morphological traits of Cheng index's method might be an alternative method to STRUCTURE for population structure analysis. A percentage of 26% of the total markers were found to detect the similar population structure as the whole SSR marker set did. Gene diversity and MRD between the two subspecies varied considerably across the genome, which might be used to identify candidate genes for the traits under domestication and artificial selection of *indica* and *japonica* rice. The percentage of SSR loci pairs in significant LD was a little low, indicating more varieties and more markers are required to raise the power to detect LD. A certain numbers of LD blocks were observed either in the entire germplasm set or in the subgroups. The average length of LD blocks varied from 2.8 to 7.1 cM, and the LD decay distance in subgroups could reduce to less than 5 cM, which indicated that fine mapping based on association mapping in the core collection might be possible.

## Supporting Information

Figure S1 **Cluster plot based Cheng's index.** Clustering analysis on the six phenotypic traits of Cheng's index as well as Cheng's index based on Ward distance.
(TIF)

Figure S2 **Delta** ***K*** **change according to different** ***K*** **among (a) the entire core collection and (b) the Subgroup 1 identified by STRUCTURE under Admixture model.**
(TIF)

Figure S3 **Principal component analysis (a), Principal coordinate analysis based on modified Roger's distance estimates (b), and LAPSTRUCT analysis on SSR marker genotypes of the entire population (c).** PC 1 and PC 2 refer to the first and second principal components or coordinates, respectively. Lap 1 and Lap 2 refer to the first and second lapvectors, respectively. The numbers in parentheses refer to the proportion of variance explained by the corresponding axes. Symbols identify the germplasm types and colors the STRUCTURE subgroups. SG 1 and SG 2 are the two subgroups identified by STRUCTURE based on the membership probability threshold of 0.80, and AD admixed. I, IC, J, JC are *indic*a, *indica*-clined, *japonica*, *japonica*-clined rice.
(TIF)

Figure S4 **Bayesian Information Criterion (BIC) against 1–30 subgroups from MCLUST.** BIC against 1–30 subgroups based on (a) all the SSR marker genotypes and (b) six traits of Cheng's index plus Cheng's index for all the varieties of entire population. EII, EEI, EVI, EEE, VEV, VII, VEI, VVI, EEV, and VVV are the models provided by MCLUST.
(TIF)

Figure S5 **Gene diversity for** ***indica*** **and** ***japonica*** **rice across the rice genome.** Red and blue lines indicate gene diversity of *indica* and *japonica* rice, respectively. Dashed lines indicate the average gene diversity of the corresponding germplasm type. Vertical lines at each point indicate standard error which was calculated by bootstrapping across genotypes. Vertical lines at the x axis indicate genetic map positions of the SSR loci on the chromosome.
(TIF)

Figure S6 **Modified Roger's distance (MRD) between** ***indica*** **and** ***japonica*** **rice across the rice genome.** Dashed lines indicate average MRD across the genome and dotted lines average MRD for each chromosome. Vertical lines at each point represent the standard error multiplied by 10 which were calculated by bootstrapping across genotypes. Vertical lines at the x axis indicate genetic map positions of the SSR loci on the chromosome.
(TIF)

Figure S7 **LD decay plot within the subgroup.** Squared correlations of allele frequencies (*r^2^*, blue triangles) and weighted standardized disequilibrium coefficient (*D*', red triangles) against genetic distance(cM) between linked loci in SG 1 (A) and SG 2 (B). The horizontal line indicates the 75th percentile of *r^2^* for unlinked loci.
(TIF)

Table S1 **Accessions, variety names, origin, germplasm types, and Cheng's index of 150 rice varieties in the core collection.** The varieties were sorted according to their STRUCTURE membership probability as Figure 1(a). *Indica* or *japonica* characteristic were identified by Cheng's index, i.e. TI, typical *indica* (1–7 score), IC, *indica*_clined (8–13 score), JC, *japonica*_clined (14–17 score), and TJ, typical *japonica* (18–24 score). E represents early seasonal, L late seasonal rice. S represents waxy rice and N non-waxy rice. Cheng's index was based on the score of the six phenotypic traits for each variety.
(DOC)

Table S2 **Summary statistics of the 274 SSR markers used in this study.** Note: Chr No-Chromosome number, AN-number of alleles per locus, GD-Gene diversity, PIC-Polymorphism information content.
(DOC)

## References

[1] Li Xl, Lu Yg, Li Jq, Xu Hm, Muhammad QS (2011). Strategies on Sample Size Determination and Qualitative and Quantitative Traits Integration to Construct Core Collection of Rice (*Oryza sativa*).. Rice Science.

[2] Londo JP, Chiang YC, Hung KH, Chiang TY, Schaal BA (2006). Phylogeography of Asian wild rice, *Oryza rufipogon*, reveals multiple independent domestications of cultivated rice, *Oryza sativa*.. Proc Natl Acad Sci U S A.

[3] Remington DL, Thornsberry JM, Matsuoka Y, Wilson LM, Whitt SR (2001). Structure of linkage disequilibrium and phenotypic associations in the maize genome.. Proc Natl Acad Sci U S A.

[4] Thornsberry JM, Goodman MM, Doebley J, Kresovich S, Nielsen D (2001). Dwarf8 polymorphisms associate with variation in flowering time.. Nat Genet.

[5] Stich B, Piepho HP, Schulz B, Melchinger AE (2008). Multi-trait association mapping in sugar beet (*Beta vulgaris* L.).. Theoretical and Applied Genetics.

[6] Abdurakhmonov IY, Abdukarimov A (2008). Application of association mapping to understanding the genetic diversity of plant germplasm resources.. Int J Plant Genomics.

[7] Flint-Garcia SA, Thuillet AC, Yu JM, Pressoir G, Romero SM (2005). Maize association population: a high-resolution platform for quantitative trait locus dissection.. Plant J.

[8] Frankel OH (1984). Genetic perspectives of germplasm conservation..

[9] Frankel OH, Brown AHD (1984a). Genetics: New frontiers..

[10] Frankel OH, Brown AHD (1984b). Crop genetics resurces conservation and evaluation..

[11] Yu P, Li Z, Zhang H, Cao Y, Li D (2003). Sampling strategy of primary core collection of common wild rice (*Oryza rufipogon* Griff.) in China.. J China Agr U.

[12] Goldstein DB, Weale ME (2001). Population genomics: linkage disequilibrium holds the key.. Curr Biol.

[13] Flint-Garcia SA, Thornsberry JM, Buckler ESt (2003). Structure of linkage disequilibrium in plants.. Annu Rev Plant Biol.

[14] Breseghello F, Sorrells ME (2006). Association mapping of kernel size and milling quality in wheat (*Triticum aestivum* L.) cultivars.. Genetics.

[15] Yu JM, Pressoir G, Briggs WH, Bi IV, Yamasaki M (2006). A unified mixed-model method for association mapping that accounts for multiple levels of relatedness.. Nature Genetics.

[16] Agrama HA, Eizenga GC, Yan W (2007). Association mapping of yield and its components in rice cultivars.. Molecular Breeding.

[17] Agrama HA, Eizenga GC (2008). Molecular diversity and genome-wide linkage disequilibrium patterns in a worldwide collection of *Oryza sativa* and its wild relatives.. Euphytica.

[18] Buckler ESt, Thornsberry JM (2002). Plant molecular diversity and applications to genomics.. Curr Opin Plant Biol.

[19] Kruglyak L (1999). Prospects for whole-genome linkage disequilibrium mapping of common disease genes.. Nat Genet.

[20] Stich B, Melchinger AE, Frisch M, Maurer HP, Heckenberger M (2005). Linkage disequilibrium in European elite maize germplasm investigated with SSRs.. Theoretical and Applied Genetics.

[21] Garris AJ, Tai TH, Coburn J, Kresovich S, McCouch S (2005). Genetic structure and diversity in *Oryza sativa* L. Genetics.

[22] Zhang DL, Zhang HL, Wei XH, Qi YW, Wang MX (2007). Genetic structure and diversity of *Oryza sativa* L. in Guizhou, China.. Chinese Science Bulletin.

[23] Zhang DL, Zhang HL, Wang MX, Sun JL, Qi YW (2009). Genetic structure and differentiation of *Oryza sativa* L. in China revealed by microsatellites.. Theoretical and Applied Genetics.

[24] Jin L, Lu Y, Xiao P, Sun M, Corke H (2010). Genetic diversity and population structure of a diverse set of rice germplasm for association mapping.. Theoretical and Applied Genetics.

[25] Pritchard JK, Stephens M, Donnelly P (2000a). Inference of population structure using multilocus genotype data.. Genetics.

[26] Pearson K (1901). On lines and planes of closest fit to system of points in space.. Philosophical Magazine.

[27] Gower JC (1966). Some distance properties of latent root and vector methods used in multivariate analysis.. Biometrika.

[28] Zhang J, Niyogi P, McPeek MS (2009). Laplacian Eigenfunctions Learn Population Structure.. PLoS One.

[29] Fraley C, Raftery AE (2007). Model-based methods of classification: Using the MCLUSTt software in chemometrics.. Journal of Statistical Software.

[30] XU Zheng-Jin LJ-q, HUANG Rui-dong, JIANG Jian, CHEN Wen-fu, ZHANG Long-Bu (2005). Subspecific Characteristics and Classification of Rice Varieties Developed Through *Indica* and *Japonica* Crossing.. AGRICULTURAL SCIENCES IN CHINA.

[31] ZHU Chun-jie XH, GUO Yan-hua, WANG Jia-yu, LIU Hong-guang, XU Zheng-jin (2007). Discrimination of *indica* and *japonica* Subspecies and Variations of Vascular Bundle Characteristics in Recombinant Inbred Lines Derived from an indica/japonica Cross.. CHINESE JOURNAL OF RICE SCIENCE.

[32] Kato S (1930). On the affinity of the cultivated rice varieties of rice plants, *Oryza sativa* L. J Deptt Agri Kyushu Imp Univ.

[33] Oka HI (1974). Analysis of genes controlling F1 sterility in rice by the use of isogenic lines.. Genetics.

[34] Qanbari S, Pimentel ECG, Tetens J, Thaller G, Lichtner P (2010). A genome-wide scan for signatures of recent selection in Holstein cattle.. Animal Genetics.

[35] Garris AJ, McCouch SR, Kresovich S (2003). Population structure and its effect on haplotype diversity and linkage disequilibrium surrounding the *xa5* locus of rice (*Oryza sativa* L.).. Genetics.

[36] Olsen KM, Caicedo AL, Polato N, McClung A, McCouch S (2006). Selection under domestication: evidence for a sweep in the rice waxy genomic region.. Genetics.

[37] Mather KA, Caicedo AL, Polato NR, Olsen KM, McCouch S (2007). The extent of linkage disequilibrium in rice (*Oryza sativa* L.).. Genetics.

[38] Rakshit S, Rakshit A, Matsumura H, Takahashi Y, Hasegawa Y (2007). Large-scale DNA polymorphism study of *Oryza sativa* and *O. rufipogon* reveals the origin and divergence of Asian rice.. Theoretical and Applied Genetics.

[39] Powell W, Morgante M, Andre C, Hanafey M, Vogel J (1996). The comparison of RFLP, RAPD, AFLP and SSR (microsatellite) markers for germplasm analysis.. Mol Breeding.

[40] Stich B, Van Inghelandt D, Melchinger AE, Lebreton C (2010). Population structure and genetic diversity in a commercial maize breeding program assessed with SSR and SNP markers.. Theoretical and Applied Genetics.

[41] Zheng KL, Huang N, Bennett J (1995). PCR-based phylogenetic analysis of wide compatibility varieties in *Oryza sativa* L. Theoretical and Applied Genetics.

[42] Panaud O, Chen X, McCouch SR (1996). Development of microsatellite markers and characterization of simple sequence length polymorphism (SSLP) in rice (*Oryza sativa* L.).. Mol Gen Genet.

[43] Nei M (1987). Molecular evolutionary genetics..

[44] Wright S (1978). Evolution and genetics of populations, vol IV..

[45] Evanno G, Regnaut S, Goudet J (2005). Detecting the number of clusters of individuals using the software STRUCTURE: a simulation study.. Molecular Ecology.

[46] Rosenberg NA (2004). DISTRUCT: a program for the graphical display of population structure.. Molecular Ecology Notes.

[47] Hubisz MJ, Falush D, Stephens M, Pritchard JK (2009). Inferring weak population structure with the assistance of sample group information.. Mol Ecol Resour.

[48] Pritchard JK XW, Daniel Falush (2009). Documentation for STRUCTURE software: Version 2.3..

[49] Saitou N, Nei M (1987). The neighbor-joining method: a new method for reconstructing phylogenetic trees.. Mol Biol Evol.

[50] Tamura K, Dudley J, Nei M, Kumar S (2007). MEGA4: Molecular evolutionary genetics analysis (MEGA) software version 4.0.. Molecular Biology and Evolution.

[51] Price AL, Patterson NJ, Plenge RM, Weinblatt ME, Shadick NA (2006). Principal components analysis corrects for stratification in genome-wide association studies.. Nat Genet.

[52] Li J, Lühmann AK, Weißleder K, Stich B (2011). Genome-wide distribution of genetic diversity and linkage disequilibrium in elite sugar beet germplasm.. BMC Genomics.

[53] Liu KJ, Goodman M, Muse S, Smith JS, Buckler E (2003). Genetic structure and diversity among maize inbred lines as inferred from DNA microsatellites.. Genetics.

