# Supplementary figures and images for: Population Structure and Genetic Diversity in a Rice Core Collection (Oryza sativa L.) Investigated with SSR Markers
Source: PLoS One. 2011 Dec 2;6(12):e27565. doi: 10.1371/journal.pone.0027565 (PMC3229487; doi:10.1371/journal.pone.0027565)

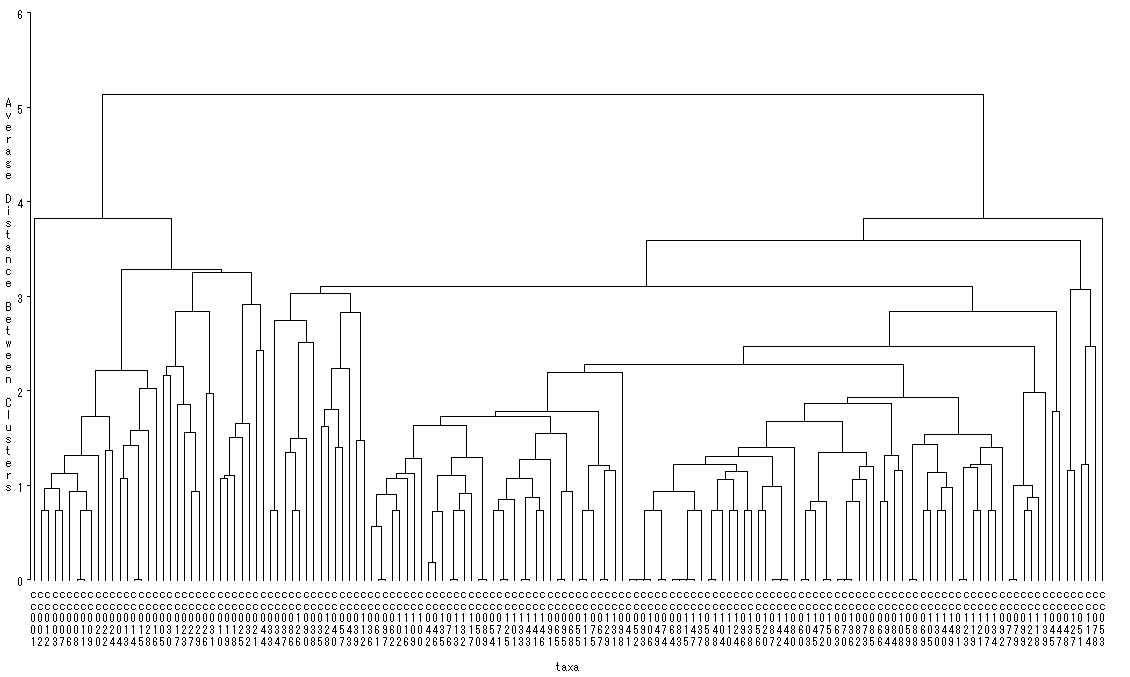

Supplement: Figure S1 — Cluster plot based Cheng's index. Clustering analysis on the six phenotypic traits of Cheng's index as well as Cheng's index based on Ward distance. (TIF) [file pone.0027565.s001.tif]

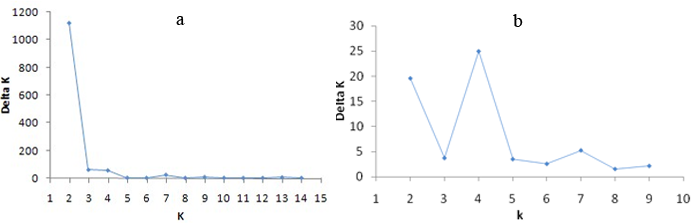

Supplement: Figure S2 — Delta K change according to different K among (a) the entire core collection and (b) the Subgroup 1 identified by STRUCTURE under Admixture model. (TIF) [file pone.0027565.s002.tif]

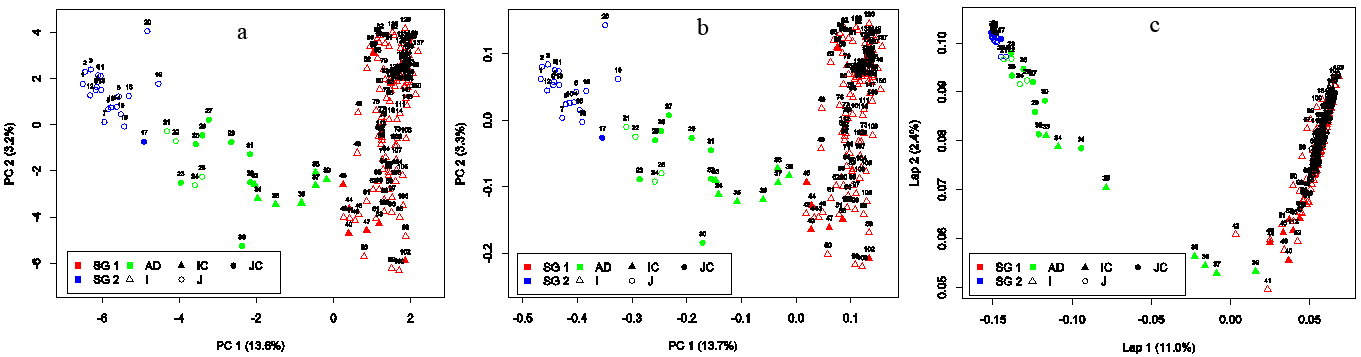

Supplement: Figure S3 — Principal component analysis (a), Principal coordinate analysis based on modified Roger's distance estimates (b), and LAPSTRUCT analysis on SSR marker genotypes of the entire population (c). PC 1 and PC 2 refer to the first and second principal components or coordinates, respectively. Lap 1 and Lap 2 refer to the first and second lapvectors, respectively. The numbers in parentheses refer to the proportion of variance explained by the corresponding axes. Symbols identify the germplasm types and colors the STRUCTURE subgroups. SG 1 and SG 2 are the two subgroups identified by STRUCTURE based on the membership probability threshold of 0.80, and AD admixed. I, IC, J, JC are indica, indica-clined, japonica, japonica-clined rice. (TIF) [file pone.0027565.s003.tif]

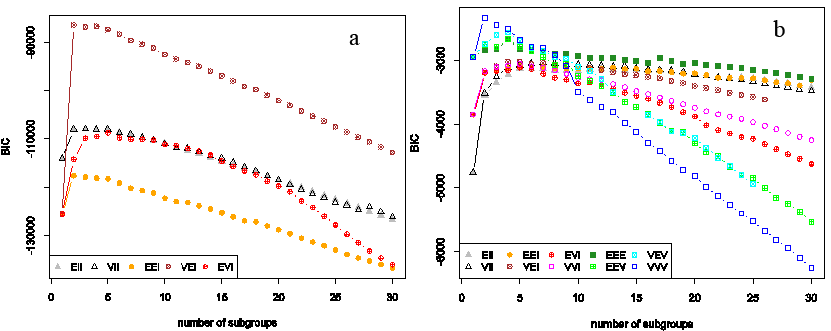

Supplement: Figure S4 — Bayesian Information Criterion (BIC) against 1–30 subgroups from MCLUST. BIC against 1–30 subgroups based on (a) all the SSR marker genotypes and (b) six traits of Cheng's index plus Cheng's index for all the varieties of entire population. EII, EEI, EVI, EEE, VEV, VII, VEI, VVI, EEV, and VVV are the models provided by MCLUST. (TIF) [file pone.0027565.s004.tif]

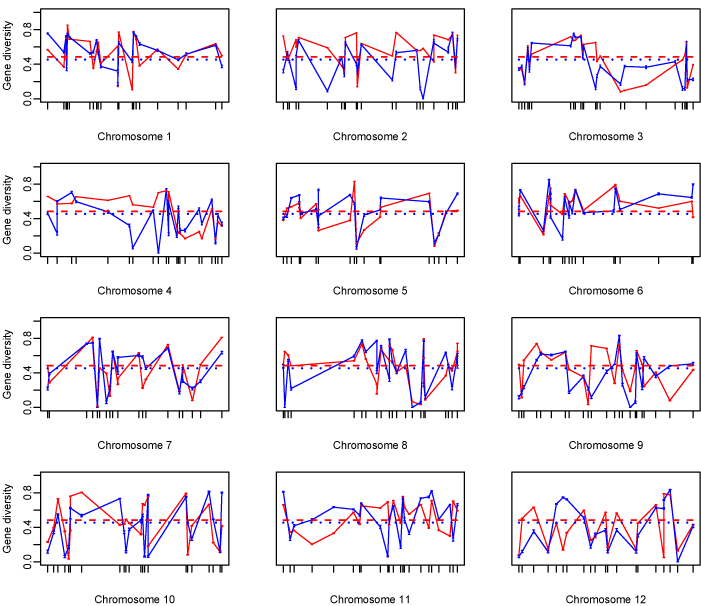

Supplement: Figure S5 — Gene diversity for indica and japonica rice across the rice genome. Red and blue lines indicate gene diversity of indica and japonica rice, respectively. Dashed lines indicate the average gene diversity of the corresponding germplasm type. Vertical lines at each point indicate standard error which was calculated by bootstrapping across genotypes. Vertical lines at the x axis indicate genetic map positions of the SSR loci on the chromosome. (TIF) [file pone.0027565.s005.tif]

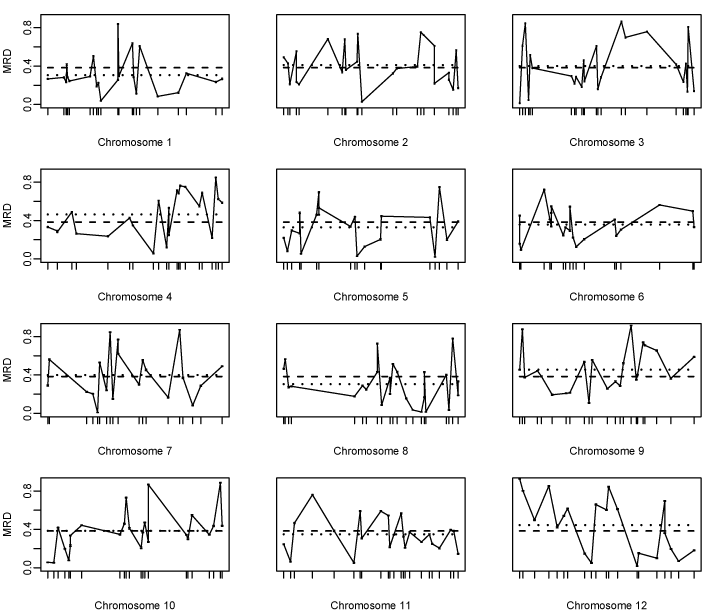

Supplement: Figure S6 — Modified Roger's distance (MRD) between indica and japonica rice across the rice genome. Dashed lines indicate average MRD across the genome and dotted lines average MRD for each chromosome. Vertical lines at each point represent the standard error multiplied by 10 which were calculated by bootstrapping across genotypes. Vertical lines at the x axis indicate genetic map positions of the SSR loci on the chromosome. (TIF) [file pone.0027565.s006.tif]

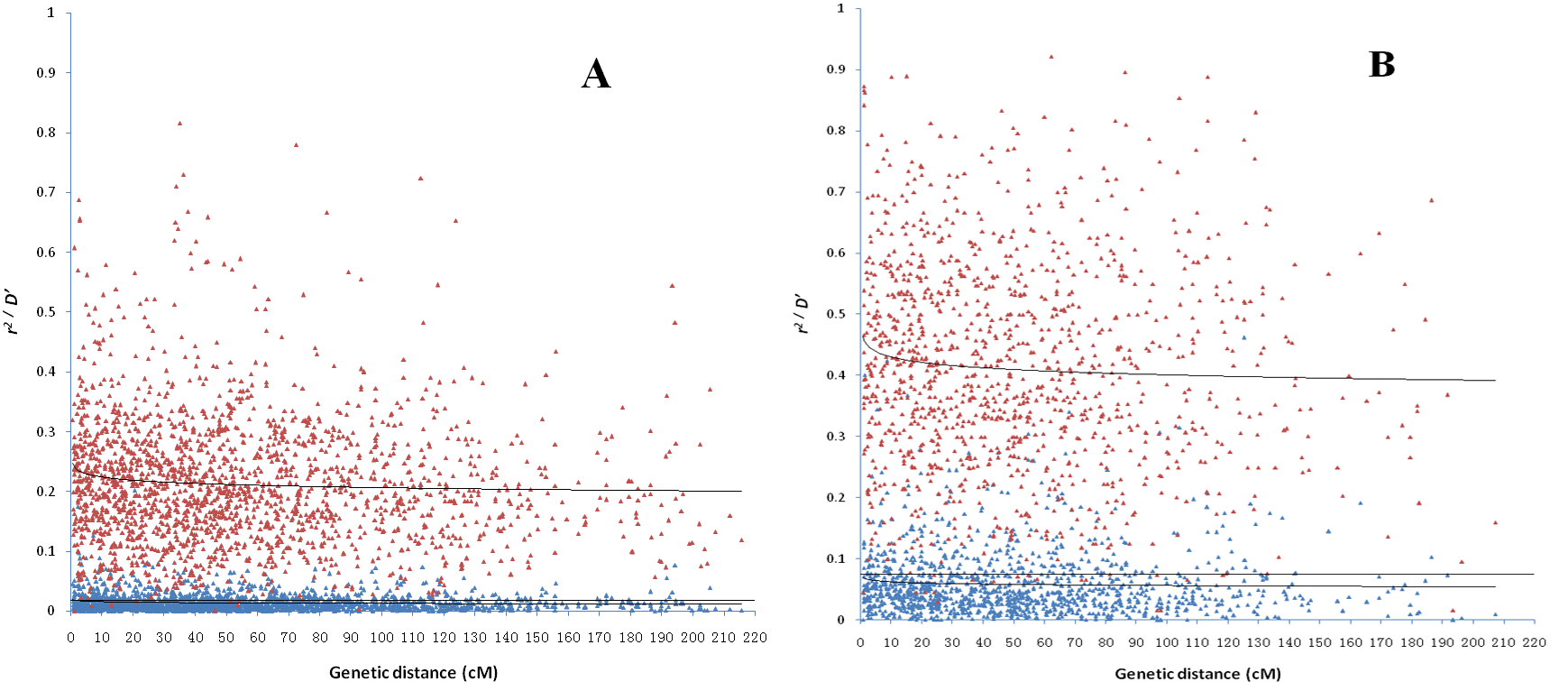

Supplement: Figure S7 — LD decay plot within the subgroup. Squared correlations of allele frequencies (r2, blue triangles) and weighted standardized disequilibrium coefficient (D', red triangles) against genetic distance(cM) between linked loci in SG 1 (A) and SG 2 (B). The horizontal line indicates the 75th percentile of r2 for unlinked loci. (TIF) [file pone.0027565.s007.tif]
