# Supplementary material for: Population Structure and Genetic Diversity in a Rice Core Collection (Oryza sativa L.) Investigated with SSR Markers
Source: PLoS One. 2011 Dec 2;6(12):e27565. doi: 10.1371/journal.pone.0027565 (PMC3229487; doi:10.1371/journal.pone.0027565)
Supplement: Table S1 — Accessions, variety names, origin, germplasm types, and Cheng's index of 150 rice varieties in the core collection. The varieties were sorted according to their STRUCTURE membership probability as Figure 1(a). Indica or japonica characteristic were identified by Cheng's index, i.e. TI, typical indica (1–7 score), IC, indica_clined (8–13 score), JC, japonica_clined (14–17 score), and TJ, typical japonica (18–24 score). E represents early seasonal, L late seasonal rice. S represents waxy rice and N non-waxy rice. Cheng's index was based on the score of the six phenotypic traits for each variety. (DOC) [file pone.0027565.s008.doc]

| **Acc.** | **Variety name** | **Origin** | ***Indica* vs.**  ***Japonica*** | **Seasonal** | **Waxy** | **Cheng's index** |
| --- | --- | --- | --- | --- | --- | --- |
| CC1 | Yin guang | Japan | *TJ* | E | N | 18 |
| CC2 | Ao guo 5-B | Japan | *TJ* | E | N | 19 |
| CC3 | Ai you | Japan | *TJ* | E | N | 21 |
| CC4 | Tie geng yi shi ao | Yangtze River region | *TJ* | E | N | 21 |
| CC5 | Guo zhu | Japan | *TJ* | E | N | 20 |
| CC6 | Ben dao | North China | *TJ* | E | N | 19 |
| CC7 | Mang shui dao | Yangtze River region | *TJ* | E | N | 21 |
| CC8 | Bai mang gao li han dao bai | North China | *TJ* | E | N | 20 |
| CC9 | Jiu yue han | Northeast China | *TJ* | E | N | 19 |
| CC10 | Bi jie ma wei hong gu | Yunnan-Kweichow Plateau | *TJ* | E | N | 20 |
| CC11 | Ai da tou | Yangtze River region | *TJ* | E | N | 21 |
| CC12 | Gui zao bai he | Yangtze River region | *TJ* | E | N | 21 |
| CC13 | Xiang dao | North China | *TJ* | E | S | 20 |
| CC14 | Zi jin gu | Northeast China | *TJ* | E | N | 20 |
| CC15 | Xiang chuan | Japan | *TJ* | E | N | 20 |
| CC16 | Nagabo | Taiwan | *TJ* | L | N | 18 |
| CC17 | Bai ke da nuo | South China | *TJ* | L | S | 15 |
| CC18 | San pai zhong | South China | *TJ* | L | N | 18 |
| CC19 | Kai xuan | Japan | *TJ* | E | S | 18 |
| CC20 | Shi ban zhan | North China | *TJ* | E | N | 20 |
| CC21 | He ke da nuo | South China | *TJ* | L | S | 20 |
| CC22 | Shen shui wan dao | Yangtze River region | *TJ* | E | N | 20 |
| CC23 | Hong ben dao | Yangtze River region | *JC* | E | N | 17 |
| CC24 | Duan mang zi jin gu | Northeast China | *TJ* | E | N | 22 |
| CC25 | Bei jing jiang mi | North China | *JC* | E | S | 17 |
| CC26 | Daeri | Celebes | *JC* | L | N | 14 |
| CC27 | Jian tou nuo | South China | *JC* | L | N | 15 |
| CC28 | Long you man dao | Yangtze River region | *TJ* | E | N | 20 |
| CC29 | Kun shan zhu zhou dao | Yangtze River region | *JC* | E | N | 14 |
| CC30 | Huang ke zao 2 | Yangtze River region | *JC* | E | N | 17 |
| CC31 | Sheng fang da bai gu | North China | *JC* | E | N | 16 |
| CC32 | Xiao dou | Japan | *JC* | L | S | 17 |
| CC33 | Poetih | Celebes | *IC* | L | N | 13 |
| CC34 | Tebaro | Sumbawa | *IC* | L | N | 12 |
| CC35 | Ao hua da gui tou hong | Yangtze River region | *IC* | E | N | 9 |
| CC36 | Hui bei zi | Yunnan-Kweichow Plateau | *IC* | E | N | 11 |
| CC37 | Ba shi zi | Yangtze River region | *IC* | E | N | 9 |
| CC38 | Zao sheng da ye | Japan | *IC* | E | N | 10 |
| CC39 | Bnlastog | Low latitude region | *IC* | L | S | 13 |
| CC40 | Nuo mi | North China | *IC* | E | S | 10 |
| CC41 | Xi chuan huang liu | South China | *TI* | L | N | 6 |
| CC42 | Hei ju dao | Yangtze River region | *TI* | E | N | 8 |
| CC43 | Guang fu 1 | Taiwan | *TI* | L | N | 4 |
| CC44 | Zhong qi jia qing | Yangtze River region | *IC* | L | N | 13 |
| CC45 | Ⅲ-49-4xi chuan huang | Taiwan | *IC* | L | N | 10 |
| CC46 | Xin xian li | Yangtze River region | *TI* | E | S | 8 |
| CC47 | Da liu tiao dao | Yangtze River region | *IC* | E | S | 10 |
| CC48 | Bai ke | South China | *TI* | L | N | 2 |
| CC49 | Chuan chi 1 | Central China | *TI* | E | N | 5 |
| CC50 | Tai nong 46 | Taiwan | *TI* | L | N | 8 |
| CC51 | Ba chong sui | Japan | *TI* | E | N | 7 |
| CC52 | Yun nan bai | Central China | *TI* | L | N | 5 |
| CC53 | Liao yang ben di 4 | Northeast China | *IC* | E | N | 11 |
| CC54 | You zhan hong | South China | *TI* | L | N | 4 |
| CC55 | Hei nuo | South China | *TI* | L | N | 6 |
| CC56 | Xian zi zhan | Central China | *TI* | E | N | 3 |
| CC57 | Da tou meng | Central China | *IC* | E | N | 11 |
| CC58 | Chi bai gan zhan | Central China | *TI* | E | N | 5 |
| CC59 | Zeng cheng xiang shan zhan | South China | *TI* | E | N | 8 |
| CC60 | Cang wu shan he zhan | South China | *TI* | L | N | 4 |
| CC61 | Da gu zao | South China | *TI* | E | N | 8 |
| CC62 | Jie yang dong liao zhong | South China | *TI* | E | N | 8 |
| CC63 | Nan xiong ku gua zao | South China | *TI* | E | N | 4 |
| CC64 | Chang mang | South China | *TI* | E | N | 4 |
| CC65 | Mandi | Celebes | *TI* | L | N | 7 |
| CC66 | Bai gu | South China | *TI* | L | S | 3 |
| CC67 | Hong zao gu | Yunnan-Kweichow Plateau | *TI* | E | N | 8 |
| CC68 | Cang wu shan he zhan | South China | *TI* | E | S | 6 |
| CC69 | Zao die zhan gu | Central China | *TI* | E | N | 7 |
| CC70 | Su zhou zhan | Central China | *TI* | L | N | 4 |
| CC71 | Yang zhan 3 | South China | *TI* | L | N | 6 |
| CC72 | Luo ding zhan 1 | South China | *TI* | L | N | 5 |
| CC73 | Gen yin 29 | South China | *TI* | E | N | 5 |
| CC74 | Wu ke nuo | South China | *TI* | L | N | 5 |
| CC75 | Hua bai ke | South China | *TI* | L | N | 8 |
| CC76 | Guang ye hong mi | South China | *TI* | E | N | 7 |
| CC77 | Da nuo | South China | *TI* | L | N | 8 |
| CC78 | Bai xu | South China | *TI* | L | N | 6 |
| CC79 | Ya he | South China | *TI* | L | N | 8 |
| CC80 | Xu zai | South China | *TI* | L | N | 6 |
| CC81 | Dong an hou zi pu xiao he | Central China | *TI* | E | N | 7 |
| CC82 | Tie gu pao | Central China | *TI* | E | N | 6 |
| CC83 | Chi mao zhan | South China | *TI* | E | N | 4 |
| CC84 | Hu bei zao | Central China | *TI* | L | N | 6 |
| CC85 | Ya jing mi | South China | *TI* | L | N | 2 |
| CC86 | Ba shi zi | Central China | *TI* | E | N | 4 |
| CC87 | Dong jun zi | Central China | *TI* | L | N | 5 |
| CC88 | Early | Unknown | *TI* | L | N | 7 |
| CC89 | Nuo | South China | *TI* | L | S | 8 |
| CC90 | Gui zhao he 2 | Japan | *TI* | L | S | 4 |
| CC91 | Hei nuo | Unknown | *TI* | L | S | 5 |
| CC92 | Da yi mao | Central China | *TI* | E | N | 6 |
| CC93 | Gai cao zhan | Central China | *TI* | E | N | 6 |
| CC94 | Gamal | Unknown | *TI* | L | N | 6 |
| CC95 | Bu gou wei | South China | *TI* | L | S | 7 |
| CC96 | Bai ke xi nuo | South China | *TI* | L | S | 6 |
| CC97 | Ben dao | North China | *TI* | E | N | 8 |
| CC98 | Ba xian shu | Japan | *TI* | E | N | 6 |
| CC99 | Guang hong mi dao | Yangtze River region | *TI* | E | N | 8 |
| CC100 | Wu mang yan guo qing | North China | *TI* | E | N | 8 |
| CC101 | Chang xu nuo | South China | *TI* | L | N | 6 |
| CC102 | Jiang wan 15 | Central China | *IC* | L | S | 11 |
| CC103 | Bai hua er | South China | *TI* | E | N | 4 |
| CC104 | Liu chang xian | South China | *TI* | E | N | 7 |
| CC105 | Bai yin 3 | South China | *TI* | E | N | 7 |
| CC106 | Shui zao huang pi | South China | *TI* | E | N | 6 |
| CC107 | Yin 2 dong 7 | South China | *TI* | E | N | 7 |
| CC108 | Hou ma | South China | *TI* | E | N | 6 |
| CC109 | Dong zhu 2 | South China | *TI* | E | N | 8 |
| CC110 | Hong gen da mi | South China | *TI* | L | N | 4 |
| CC111 | Ben cheng guan yin zhan | Central China | *TI* | E | N | 7 |
| CC112 | Xi miao gu | South China | *IC* | E | N | 10 |
| CC113 | 186-zao guan yin zhan | Central China | *TI* | E | N | 8 |
| CC114 | Chang mang hei ma zao | Yunnan | *TI* | E | N | 8 |
| CC115 | Shui tian zhan gu nuo | South China | *TI* | L | N | 4 |
| CC116 | Chang han da hua ke | South China | *IC* | L | N | 12 |
| CC117 | Da he | South China | *TI* | L | N | 8 |
| CC118 | Zeng cheng hei nuo | South China | *TI* | L | N | 8 |
| CC119 | Ya he | South China | *TI* | L | N | 8 |
| CC120 | Bai gu zhan | Central China | *TI* | L | N | 5 |
| CC121 | Die zhi | South China | *TI* | L | N | 8 |
| CC122 | You zhan | South China | *TI* | L | N | 8 |
| CC123 | Chang sha wu qu wan dao | Central China | *TI* | E | N | 7 |
| CC124 | Tong ling hu nan xian | Central China | *TI* | E | N | 5 |
| CC125 | Xiao mao dao | Central China | *TI* | E | N | 5 |
| CC126 | Jing xian si qu er gan | Central China | *TI* | E | N | 6 |
| CC127 | Zi xing er qu si dou xu | Central China | *TI* | E | N | 2 |
| CC128 | Chen hui fu dao | Central China | *TI* | E | N | 6 |
| CC129 | Bai gan zi | Central China | *TI* | E | N | 8 |
| CC130 | Han lu wei zhan | Central China | *TI* | E | N | 7 |
| CC131 | Xin hua san qu tang mao zhan | Central China | *TI* | E | N | 7 |
| CC132 | Ta gu zhan | Central China | *TI* | E | N | 7 |
| CC133 | Mian tiao zhan | Central China | *TI* | E | N | 6 |
| CC134 | Hu guang zhan | Central China | *TI* | E | N | 8 |
| CC135 | Jiang an da ye zao | Central China | *TI* | E | N | 8 |
| CC136 | Tie ban zhan | Central China | *TI* | L | N | 4 |
| CC137 | Da gu zao | Central China | *TI* | L | N | 5 |
| CC138 | Ding nan dong zhan | Central China | *TI* | L | N | 5 |
| CC139 | Lao wu gu | Central China | *TI* | L | N | 6 |
| CC140 | Xin ban chang ke zi | Central China | *TI* | L | N | 6 |
| CC141 | Bai zhan gu | Central China | *TI* | L | N | 5 |
| CC142 | Si chuan zhan | Central China | *TI* | L | N | 5 |
| CC143 | Gao jiao gui hua | Central China | *TI* | L | N | 6 |
| CC144 | Chang shu wu wi dao | Central China | *TI* | L | N | 4 |
| CC145 | Da nuo bai dong | Central China | *TI* | L | N | 4 |
| CC146 | Lin chuan da ye zao | South China | *TI* | L | N | 5 |
| CC147 | Da bai cao | North China | *TI* | E | N | 3 |
| CC148 | Chang ning wu qu nan tou zhan | Central China | *TI* | E | N | 7 |
| CC149 | Pi xian da ye zi | Central China | *TI* | E | N | 8 |
| CC150 | Xi zi zhan | Central China | *TI* | L | N | 5 |
