# Supplementary material for: Population Structure and Genetic Diversity in a Rice Core Collection (Oryza sativa L.) Investigated with SSR Markers
Source: PLoS One. 2011 Dec 2;6(12):e27565. doi: 10.1371/journal.pone.0027565 (PMC3229487; doi:10.1371/journal.pone.0027565)
Supplement: Table S2 — Summary statistics of the 274 SSR markers used in this study. Note: Chr No-Chromosome number, AN-number of alleles per locus, GD-Gene diversity, PIC-Polymorphism information content. (DOC) [file pone.0027565.s009.doc]

| **locus** | **Chr No.** | **position**  **(cM)** | **A N** | **GD** | **PIC** |  | **locus** | **Chr No.** | **position**  **(cM)** | **AN** | **GD** | **PIC** |
| --- | --- | --- | --- | --- | --- | --- | --- | --- | --- | --- | --- | --- |
| PSM41 | 1 | 10.9 | 6.0000 | 0.6319 | 0.5944 |  | RM51 | 7 | 0 | 2.0000 | 0.4570 | 0.3526 |
| RM84 | 1 | 26.2 | 5.0000 | 0.4316 | 0.3981 |  | RM427 | 7 | 1.1 | 2.0000 | 0.4146 | 0.3286 |
| RM220 | 1 | 28.4 | 5.0000 | 0.7251 | 0.6805 |  | PSM142 | 7 | 26 | 5.0000 | 0.7519 | 0.7108 |
| RM348-1 | 1 | 28.9 | 2.0000 | 0.4956 | 0.3728 |  | RM180 | 7 | 30.1 | 9.0000 | 0.8128 | 0.7893 |
| RM1 | 1 | 29.7 | 10.0000 | 0.8588 | 0.8426 |  | RM325B | 7 | 33.3 | 2.0000 | 0.0132 | 0.0132 |
| RM283 | 1 | 31.4 | 4.0000 | 0.7162 | 0.6663 |  | RM214 | 7 | 34.7 | 6.0000 | 0.6218 | 0.5837 |
| RM576 | 1 | 51 | 3.0000 | 0.6638 | 0.5897 |  | RM445 | 7 | 39.3 | 2.0000 | 0.3432 | 0.2843 |
| RM259 | 1 | 54.2 | 3.0000 | 0.4783 | 0.4298 |  | RM125 | 7 | 41.7 | 4.0000 | 0.3962 | 0.3368 |
| RM243 | 1 | 57.3 | 5.0000 | 0.5885 | 0.5075 |  | RM432 | 7 | 43.5 | 3.0000 | 0.6589 | 0.5850 |
| RM583 | 1 | 58.9 | 4.0000 | 0.6449 | 0.5713 |  | RM11 | 7 | 47 | 4.0000 | 0.4948 | 0.4546 |
| RM577 | 1 | 61.3 | 2.0000 | 0.4032 | 0.3219 |  | RM346 | 7 | 47 | 4.0000 | 0.5190 | 0.4537 |
| RM81A | 1 | 77.5 | 3.0000 | 0.5704 | 0.5042 |  | RM182 | 7 | 61 | 7.0000 | 0.6560 | 0.5992 |
| RM129 | 1 | 77.5 | 3.0000 | 0.3847 | 0.3203 |  | RM10 | 7 | 63.5 | 3.0000 | 0.4066 | 0.3538 |
| RM562 | 1 | 78.4 | 6.0000 | 0.7738 | 0.7384 |  | RM455 | 7 | 65.7 | 2.0000 | 0.4200 | 0.3318 |
| PSM368 | 1 | 91.2 | 2.0000 | 0.3102 | 0.2661 |  | PSM336 | 7 | 80.5 | 5.0000 | 0.7286 | 0.6806 |
| RM9 | 1 | 92.4 | 6.0000 | 0.8047 | 0.7764 |  | RM234 | 7 | 88.2 | 4.0000 | 0.4387 | 0.3767 |
| RM5 | 1 | 94.9 | 4.0000 | 0.6980 | 0.6429 |  | RM18 | 7 | 90.4 | 2.0000 | 0.4644 | 0.3566 |
| RM306 | 1 | 98.1 | 6.0000 | 0.5593 | 0.5266 |  | RM47 | 7 | 90.4 | 2.0000 | 0.4992 | 0.3746 |
| RM237 | 1 | 115.2 | 3.0000 | 0.5727 | 0.4955 |  | RM429 | 7 | 96.9 | 2.0000 | 0.1128 | 0.1064 |
| RM128 | 1 | 134.8 | 2.0000 | 0.3716 | 0.3026 |  | PSM147 | 7 | 102.3 | 2.0000 | 0.4850 | 0.3674 |
| PSM334 | 1 | 142.4 | 4.0000 | 0.5480 | 0.4452 |  | RM248 | 7 | 116.6 | 10.0000 | 0.8547 | 0.8380 |
| PSM369 | 1 | 170.4 | 3.0000 | 0.6528 | 0.5786 |  | RM408 | 8 | 0 | 3.0000 | 0.5663 | 0.4832 |
| PSM370 | 1 | 176.3 | 2.0000 | 0.4968 | 0.3734 |  | RM337 | 8 | 1.1 | 3.0000 | 0.6175 | 0.5429 |
| RM109 | 2 | 0 | 5.0000 | 0.7206 | 0.6698 |  | PSM392 | 8 | 3.6 | 4.0000 | 0.6182 | 0.5474 |
| RM154 | 2 | 4.8 | 3.0000 | 0.5354 | 0.4588 |  | RM407 | 8 | 5.7 | 2.0000 | 0.4530 | 0.3504 |
| RM110 | 2 | 6.9 | 2.0000 | 0.4992 | 0.3746 |  | RM25 | 8 | 52.2 | 5.0000 | 0.5614 | 0.5166 |
| RM211 | 2 | 14.4 | 4.0000 | 0.6546 | 0.5849 |  | RM547 | 8 | 58.1 | 7.0000 | 0.7728 | 0.7429 |
| RM236 | 2 | 14.4 | 4.0000 | 0.5987 | 0.5306 |  | RM404 | 8 | 60.9 | 4.0000 | 0.6002 | 0.5253 |
| RM279 | 2 | 17.3 | 5.0000 | 0.7174 | 0.6681 |  | RM44 | 8 | 69 | 5.0000 | 0.4406 | 0.4171 |
| RM71 | 2 | 49.8 | 4.0000 | 0.6404 | 0.5716 |  | RM331 | 8 | 69 | 5.0000 | 0.3890 | 0.3395 |
| RM27 | 2 | 66 | 2.0000 | 0.4414 | 0.3440 |  | RM339 | 8 | 72.2 | 5.0000 | 0.6811 | 0.6273 |
| RM29 | 2 | 68.9 | 3.0000 | 0.4727 | 0.3885 |  | RM42 | 8 | 78.4 | 3.0000 | 0.5384 | 0.4445 |
| RM262 | 2 | 70.2 | 4.0000 | 0.7402 | 0.6921 |  | RM342A | 8 | 78.4 | 6.0000 | 0.7429 | 0.7043 |
| RM341 | 2 | 82.7 | 5.0000 | 0.7525 | 0.7133 |  | RM223 | 8 | 80.5 | 5.0000 | 0.7280 | 0.6871 |
| PSM374 | 2 | 83.6 | 2.0000 | 0.3578 | 0.2938 |  | RM284 | 8 | 83.7 | 2.0000 | 0.4712 | 0.3602 |
| PSM122 | 2 | 88.2 | 3.0000 | 0.6383 | 0.5634 |  | RM210 | 8 | 90.3 | 6.0000 | 0.5322 | 0.4838 |
| RM106 | 2 | 123.2 | 2.0000 | 0.4712 | 0.3602 |  | RM419 | 8 | 95.2 | 2.0000 | 0.0526 | 0.0512 |
| RM263 | 2 | 127.5 | 5.0000 | 0.7657 | 0.7270 |  | RM256 | 8 | 101.5 | 2.0000 | 0.0392 | 0.0384 |
| RM450 | 2 | 150.8 | 4.0000 | 0.6135 | 0.5659 |  | RM80 | 8 | 103.7 | 9.0000 | 0.8004 | 0.7747 |
| RM6 | 2 | 154.7 | 3.0000 | 0.6556 | 0.5812 |  | RM149 | 8 | 103.7 | 6.0000 | 0.5680 | 0.4738 |
| RM240 | 2 | 158 | 3.0000 | 0.6335 | 0.5572 |  | RM308 | 8 | 104.8 | 3.0000 | 0.0962 | 0.0940 |
| RM250 | 2 | 170.1 | 5.0000 | 0.6033 | 0.5684 |  | PSM351 | 8 | 119.9 | 3.0000 | 0.4794 | 0.4176 |
| RM530 | 2 | 170.1 | 4.0000 | 0.7339 | 0.6849 |  | RM458 | 8 | 121.8 | 2.0000 | 0.4928 | 0.3714 |
| RM213 | 2 | 186.4 | 4.0000 | 0.6953 | 0.6413 |  | RM447 | 8 | 124.6 | 3.0000 | 0.5848 | 0.5200 |
| RM208 | 2 | 186.4 | 4.0000 | 0.6845 | 0.6258 |  | RM281 | 8 | 128.6 | 5.0000 | 0.6522 | 0.6121 |
| RM207 | 2 | 191.2 | 6.0000 | 0.7572 | 0.7174 |  | RM264 | 8 | 128.6 | 5.0000 | 0.7217 | 0.6775 |
| RM498 | 2 | 194.6 | 2.0000 | 0.4252 | 0.3348 |  | RM296 | 9 | 0 | 2.0000 | 0.4872 | 0.3685 |
| RM138 | 2 | 196.8 | 4.0000 | 0.7359 | 0.6869 |  | RM316 | 9 | 1.8 | 3.0000 | 0.3834 | 0.3276 |
| RM60 | 3 | 0 | 2.0000 | 0.3539 | 0.2913 |  | RM444 | 9 | 3.3 | 6.0000 | 0.5284 | 0.4383 |
| RM104 | 3 | 3.5 | 3.0000 | 0.4676 | 0.4096 |  | RM219 | 9 | 11.7 | 5.0000 | 0.7651 | 0.7275 |
| RM132 | 3 | 7 | 3.0000 | 0.4228 | 0.3707 |  | RM342B | 9 | 14.3 | 4.0000 | 0.6865 | 0.6356 |
| RM22 | 3 | 11 | 4.0000 | 0.6071 | 0.5405 |  | PSM399 | 9 | 21.4 | 3.0000 | 0.5742 | 0.4866 |
| RM348-2 | 3 | 13 | 2.0000 | 0.4772 | 0.3634 |  | PSM157 | 9 | 30.6 | 4.0000 | 0.6566 | 0.6104 |
| RM231 | 3 | 15.7 | 4.0000 | 0.5947 | 0.5229 |  | PSM158 | 9 | 33 | 4.0000 | 0.3985 | 0.3493 |
| RM7 | 3 | 64 | 4.0000 | 0.7252 | 0.6748 |  | PSM160 | 9 | 42.5 | 2.0000 | 0.4509 | 0.3493 |
| RM218 | 3 | 67.8 | 6.0000 | 0.7068 | 0.6556 |  | RM409 | 9 | 45.6 | 2.0000 | 0.0768 | 0.0739 |
| PSM377 | 3 | 69.7 | 4.0000 | 0.7128 | 0.6607 |  | RM566 | 9 | 47.7 | 5.0000 | 0.6918 | 0.6380 |
| RM232 | 3 | 76.7 | 4.0000 | 0.7430 | 0.6952 |  | RM434 | 9 | 57.7 | 4.0000 | 0.6484 | 0.6007 |
| RM251 | 3 | 79.1 | 7.0000 | 0.6074 | 0.5720 |  | PSM337 | 9 | 63 | 2.0000 | 0.3648 | 0.2983 |
| PSM379 | 3 | 80 | 3.0000 | 0.6463 | 0.5732 |  | RM257 | 9 | 66.1 | 7.0000 | 0.7774 | 0.7441 |
| RM16 | 3 | 94.9 | 4.0000 | 0.6619 | 0.5920 |  | PSM338 | 9 | 68.2 | 2.0000 | 0.4911 | 0.3705 |
| PSM128 | 3 | 96.6 | 2.0000 | 0.4048 | 0.3229 |  | RM242 | 9 | 73.3 | 4.0000 | 0.4291 | 0.3575 |
| RM282 | 3 | 100.6 | 2.0000 | 0.4986 | 0.3743 |  | RM553 | 9 | 76.7 | 2.0000 | 0.4252 | 0.3348 |
| RM156 | 3 | 125.7 | 2.0000 | 0.3506 | 0.2891 |  | RM278 | 9 | 77.5 | 4.0000 | 0.7036 | 0.6474 |
| PSM130 | 3 | 130.7 | 2.0000 | 0.3200 | 0.2688 |  | RM201 | 9 | 81.2 | 3.0000 | 0.6239 | 0.5516 |
| RM135 | 3 | 157.3 | 3.0000 | 0.3954 | 0.3427 |  | RM160 | 9 | 82.4 | 8.0000 | 0.4815 | 0.4560 |
| RM293 | 3 | 193.4 | 2.0000 | 0.4608 | 0.3546 |  | PSM340 | 9 | 90.1 | 3.0000 | 0.5407 | 0.4807 |
| RM468 | 3 | 202.3 | 3.0000 | 0.3976 | 0.3474 |  | RM215 | 9 | 99.4 | 2.0000 | 0.2112 | 0.1889 |
| RM571 | 3 | 205.4 | 3.0000 | 0.4852 | 0.3738 |  | RM205 | 9 | 114.7 | 5.0000 | 0.5693 | 0.5237 |
| RM143 | 3 | 207.3 | 3.0000 | 0.6539 | 0.5797 |  | RM222 | 10 | 11.3 | 3.0000 | 0.2063 | 0.1945 |
| RM130 | 3 | 208.2 | 2.0000 | 0.3648 | 0.2983 |  | RM244 | 10 | 15 | 2.0000 | 0.3848 | 0.3108 |
| RM565 | 3 | 215.5 | 2.0000 | 0.3645 | 0.2981 |  | RM216 | 10 | 17.6 | 5.0000 | 0.7500 | 0.7082 |
| RM307 | 4 | 0 | 3.0000 | 0.6516 | 0.5765 |  | PSM163 | 10 | 21.8 | 2.0000 | 0.3036 | 0.2575 |
| RM401 | 4 | 8.5 | 3.0000 | 0.5464 | 0.4855 |  | PSM164 | 10 | 24.2 | 2.0000 | 0.0644 | 0.0624 |
| RM551 | 4 | 8.5 | 4.0000 | 0.6026 | 0.5592 |  | RM239 | 10 | 25.2 | 2.0000 | 0.4090 | 0.3253 |
| RM335 | 4 | 21.5 | 7.0000 | 0.6846 | 0.6554 |  | RM311 | 10 | 25.2 | 5.0000 | 0.7690 | 0.7318 |
| RM518 | 4 | 25.5 | 3.0000 | 0.6644 | 0.5903 |  | PSM166 | 10 | 32.1 | 6.0000 | 0.8135 | 0.7868 |
| RM471 | 4 | 53.8 | 3.0000 | 0.6016 | 0.5237 |  | PSM167 | 10 | 55.6 | 4.0000 | 0.5336 | 0.4921 |
| RM564 | 4 | 73.1 | 3.0000 | 0.6522 | 0.5788 |  | RM184 | 10 | 58.3 | 2.0000 | 0.4872 | 0.3685 |
| RM119 | 4 | 76.1 | 4.0000 | 0.4947 | 0.4332 |  | RM271 | 10 | 59.4 | 5.0000 | 0.5902 | 0.5243 |
| RM273 | 4 | 94.4 | 3.0000 | 0.5255 | 0.4127 |  | PSM169 | 10 | 61.4 | 2.0000 | 0.4892 | 0.3696 |
| RM252 | 4 | 99 | 5.0000 | 0.6750 | 0.6168 |  | PSM170 | 10 | 68.6 | 2.0000 | 0.3682 | 0.3004 |
| RM241 | 4 | 106.2 | 4.0000 | 0.7331 | 0.6845 |  | RM269 | 10 | 69.6 | 4.0000 | 0.6891 | 0.6314 |
| PSM102 | 4 | 108.2 | 4.0000 | 0.7062 | 0.6537 |  | RM258 | 10 | 70.8 | 4.0000 | 0.6015 | 0.5375 |
| RM451 | 4 | 115.5 | 2.0000 | 0.4422 | 0.3444 |  | RM304 | 10 | 73 | 6.0000 | 0.7842 | 0.7509 |
| RM303 | 4 | 116.9 | 5.0000 | 0.4532 | 0.4142 |  | RM171 | 10 | 73 | 2.0000 | 0.4090 | 0.3253 |
| RM317 | 4 | 118.3 | 3.0000 | 0.4316 | 0.3794 |  | RM228 | 10 | 96.3 | 12.0000 | 0.8227 | 0.8023 |
| PSM382 | 4 | 122.9 | 2.0000 | 0.3783 | 0.3068 |  | RM484 | 10 | 97.3 | 2.0000 | 0.1906 | 0.1724 |
| RM255 | 4 | 135.4 | 4.0000 | 0.4062 | 0.3603 |  | RM147 | 10 | 99.8 | 2.0000 | 0.4850 | 0.3674 |
| RM348 | 4 | 137.9 | 2.0000 | 0.3648 | 0.2983 |  | RM333 | 10 | 110.4 | 7.0000 | 0.7355 | 0.6939 |
| RM349 | 4 | 146.8 | 3.0000 | 0.5491 | 0.4772 |  | RM496 | 10 | 113 | 2.0000 | 0.3432 | 0.2843 |
| RM127 | 4 | 150.1 | 3.0000 | 0.4008 | 0.3256 |  | RM590 | 10 | 117.2 | 3.0000 | 0.3796 | 0.3170 |
| RM280 | 4 | 152.3 | 3.0000 | 0.5108 | 0.4560 |  | RM591 | 10 | 118.3 | 8.0000 | 0.5627 | 0.5270 |
| RM559 | 4 | 155.8 | 2.0000 | 0.4352 | 0.3405 |  | RM286 | 11 | 0 | 7.0000 | 0.7124 | 0.6705 |
| RM122 | 5 | 0 | 4.0000 | 0.4026 | 0.3794 |  | PSM171 | 11 | 4.8 | 2.0000 | 0.3279 | 0.2741 |
| RM153 | 5 | 3 | 3.0000 | 0.5032 | 0.4364 |  | PSM172 | 11 | 7.6 | 2.0000 | 0.4466 | 0.3469 |
| PSM341 | 5 | 6.5 | 3.0000 | 0.5876 | 0.5156 |  | RM167 | 11 | 20.3 | 4.0000 | 0.4573 | 0.4225 |
| PSM8 | 5 | 13 | 4.0000 | 0.6225 | 0.5498 |  | PSM410 | 11 | 35.6 | 3.0000 | 0.4451 | 0.3975 |
| PSM60 | 5 | 13 | 4.0000 | 0.6159 | 0.5596 |  | PSM411 | 11 | 49.6 | 3.0000 | 0.5810 | 0.5055 |
| PSM202 | 5 | 14.1 | 3.0000 | 0.4163 | 0.3400 |  | RM202 | 11 | 54 | 3.0000 | 0.5749 | 0.5096 |
| RM413 | 5 | 26.7 | 5.0000 | 0.6213 | 0.5770 |  | RM536 | 11 | 55.1 | 4.0000 | 0.6858 | 0.6333 |
| RM267 | 5 | 28.6 | 4.0000 | 0.4620 | 0.3989 |  | RM287 | 11 | 68.6 | 6.0000 | 0.6953 | 0.6510 |
| RM405 | 5 | 28.6 | 5.0000 | 0.6222 | 0.5652 |  | RM209 | 11 | 73.9 | 5.0000 | 0.6604 | 0.6022 |
| RM548 | 5 | 28.6 | 2.0000 | 0.3944 | 0.3167 |  | PSM416 | 11 | 74.9 | 2.0000 | 0.4608 | 0.3546 |
| RM249 | 5 | 54.3 | 7.0000 | 0.4787 | 0.4370 |  | RM229 | 11 | 77.8 | 5.0000 | 0.7360 | 0.6887 |
| RM169 | 5 | 57.9 | 8.0000 | 0.8393 | 0.8187 |  | RM457 | 11 | 83 | 2.0000 | 0.4968 | 0.3734 |
| RM516 | 5 | 59.6 | 2.0000 | 0.1010 | 0.0959 |  | PSM365 | 11 | 84.6 | 6.0000 | 0.7857 | 0.7545 |
| RM509 | 5 | 65.8 | 3.0000 | 0.3111 | 0.2882 |  | RM21 | 11 | 85.7 | 6.0000 | 0.6186 | 0.5824 |
| RM146 | 5 | 78.7 | 2.0000 | 0.4488 | 0.3481 |  | PSM366 | 11 | 89 | 4.0000 | 0.5500 | 0.4477 |
| RM164 | 5 | 79.4 | 4.0000 | 0.6215 | 0.5763 |  | PSM417 | 11 | 97.3 | 5.0000 | 0.7033 | 0.6485 |
| RM31 | 5 | 118.8 | 4.0000 | 0.7349 | 0.6862 |  | RM206 | 11 | 102.9 | 9.0000 | 0.5073 | 0.4886 |
| PSM386 | 5 | 123 | 2.0000 | 0.0890 | 0.0850 |  | PSM418 | 11 | 104.9 | 6.0000 | 0.7546 | 0.7175 |
| RM274 | 5 | 126.6 | 2.0000 | 0.4090 | 0.3253 |  | RM254 | 11 | 110 | 3.0000 | 0.4068 | 0.3293 |
| RM538 | 5 | 132.7 | 2.0000 | 0.4978 | 0.3739 |  | PSM176 | 11 | 117.9 | 3.0000 | 0.4282 | 0.3815 |
| RM334 | 5 | 141.8 | 7.0000 | 0.5857 | 0.5481 |  | RM224 | 11 | 120.1 | 5.0000 | 0.6621 | 0.6103 |
| RM469 | 6 | 2.2 | 3.0000 | 0.6593 | 0.5853 |  | RM144 | 11 | 123.2 | 5.0000 | 0.6143 | 0.5540 |
| RM170 | 6 | 2.2 | 5.0000 | 0.5832 | 0.5258 |  | RM20A | 12 | 3.2 | 2.0000 | 0.3648 | 0.2983 |
| RM589 | 6 | 3.2 | 5.0000 | 0.6827 | 0.6177 |  | RM4A | 12 | 5.2 | 3.0000 | 0.6374 | 0.5632 |
| RM510 | 6 | 20.8 | 2.0000 | 0.4002 | 0.3201 |  | PSM183 | 12 | 12.2 | 3.0000 | 0.6562 | 0.5820 |
| RM204 | 6 | 25.1 | 10.0000 | 0.7537 | 0.7297 |  | RM19 | 12 | 20.9 | 2.0000 | 0.3911 | 0.3146 |
| RM217 | 6 | 26.2 | 5.0000 | 0.7761 | 0.7417 |  | PSM184 | 12 | 26 | 4.0000 | 0.5528 | 0.4877 |
| RM225 | 6 | 26.2 | 4.0000 | 0.6719 | 0.6147 |  | PSM419 | 12 | 30 | 5.0000 | 0.3674 | 0.3475 |
| RM584 | 6 | 26.2 | 3.0000 | 0.6282 | 0.5554 |  | RM247 | 12 | 32.3 | 8.0000 | 0.5462 | 0.5264 |
| RM111 | 6 | 35.3 | 2.0000 | 0.4090 | 0.3253 |  | PSM420 | 12 | 42.7 | 3.0000 | 0.5852 | 0.4982 |
| RM253 | 6 | 37 | 5.0000 | 0.7147 | 0.6615 |  | RM179 | 12 | 46.8 | 2.0000 | 0.2351 | 0.2075 |
| RM402 | 6 | 40.3 | 3.0000 | 0.5848 | 0.4948 |  | RM101 | 12 | 49.5 | 3.0000 | 0.4344 | 0.3457 |
| RM276 | 6 | 40.3 | 11.0000 | 0.6756 | 0.6451 |  | PSM421 | 12 | 55.9 | 3.0000 | 0.6500 | 0.5746 |
| RM557 | 6 | 41.9 | 2.0000 | 0.4967 | 0.3734 |  | RM277 | 12 | 57.2 | 2.0000 | 0.3972 | 0.3183 |
| RM549 | 6 | 42.7 | 3.0000 | 0.5930 | 0.5225 |  | RM519 | 12 | 62.6 | 3.0000 | 0.6479 | 0.5722 |
| RM539 | 6 | 45.1 | 4.0000 | 0.7428 | 0.6950 |  | RM309 | 12 | 74.5 | 3.0000 | 0.1384 | 0.1334 |
| RM136 | 6 | 51.2 | 3.0000 | 0.5039 | 0.3835 |  | RM463 | 12 | 75.5 | 2.0000 | 0.4252 | 0.3348 |
| RM3 | 6 | 74.3 | 6.0000 | 0.7678 | 0.7323 |  | PSM188 | 12 | 86.5 | 3.0000 | 0.6563 | 0.5825 |
| RM541 | 6 | 75.5 | 7.0000 | 0.8038 | 0.7753 |  | RM270 | 12 | 91.3 | 4.0000 | 0.3331 | 0.3118 |
| PSM138 | 6 | 79 | 3.0000 | 0.6115 | 0.5376 |  | RM235 | 12 | 91.3 | 7.0000 | 0.8146 | 0.7900 |
| RM162 | 6 | 108.3 | 5.0000 | 0.6596 | 0.6013 |  | PSM190 | 12 | 95.1 | 8.0000 | 0.8011 | 0.7760 |
| RM340 | 6 | 133.5 | 5.0000 | 0.6920 | 0.6561 |  | PSM191 | 12 | 99.7 | 2.0000 | 0.1010 | 0.0959 |
| RM400 | 6 | 134.5 | 9.0000 | 0.5338 | 0.5066 |  | RM17 | 12 | 109.1 | 3.0000 | 0.4367 | 0.3703 |
| **Total alleles**  **Mean** |  |  |  |  |  |  |  |  |  | **1063**  **3.8796** | **0.5436** | **0.4831** |
